# Parallel multi-swarm cooperative particle swarm optimization for protein–ligand docking and virtual screening

**DOI:** 10.1186/s12859-022-04711-0

**Published:** 2022-05-30

**Authors:** Chao Li, Jinxing Li, Jun Sun, Li Mao, Vasile Palade, Bilal Ahmad

**Affiliations:** 1 Department of Computer Science and Technology, Jiangnan University, No.1800, Lihu Avenue, Wuxi, Jiangsu People’s Republic of China; 2 Centre for Computational Science and Mathematical Modelling, Coventry University, Priory Street, Coventry, CV1 5FB UK

**Keywords:** Protein–ligand docking, Virtual screening, Random drift particle swarm optimization, Autodock Vina

## Abstract

**Background:**

A high-quality docking method tends to yield multifold gains with half pains for the new drug development. Over the past few decades, great efforts have been made for the development of novel docking programs with great efficiency and intriguing accuracy. AutoDock Vina (Vina) is one of these achievements with improved speed and accuracy compared to AutoDock4. Since it was proposed, some of its variants, such as PSOVina and GWOVina, have also been developed. However, for all these docking programs, there is still large room for performance improvement.

**Results:**

In this work, we propose a parallel multi-swarm cooperative particle swarm model, in which one master swarm and several slave swarms mutually cooperate and co-evolve. Our experiments show that multi-swarm programs possess better docking robustness than PSOVina. Moreover, the multi-swarm program based on random drift PSO can achieve the best highest accuracy of protein–ligand docking, an outstanding enrichment effect for drug-like activate compounds, and the second best AUC screening accuracy among all the compared docking programs, but with less computation consumption than most of the other docking programs.

**Conclusion:**

The proposed multi-swarm cooperative model is a novel algorithmic modeling suitable for protein–ligand docking and virtual screening. Owing to the existing coevolution between the master and the slave swarms, this model in parallel generates remarkable docking performance. The source code can be freely downloaded from https://github.com/li-jin-xing/MPSOVina.

**Supplementary Information:**

The online version contains supplementary material available at 10.1186/s12859-022-04711-0.

## Background

Docking tools for predictive modeling are widely used for building candidate protein–ligand complexes, the pocket location of a specific protein and the synthesis guidance of lead compound in the field of biochemical computation. These applications of docking tools benefit from two primary docking practices: protein–ligand docking and virtual screening [1]. The protein–ligand docking illustrates how a ligand may bind, and it gives the prediction of the pose for one or several ligands binding to a given macromolecule. With regards to whether a ligand can bind, it involves the virtual screening [2], which docks, ranks and refines an extensive library of compounds against a specific target to determine their worthiness as drug candidates.

Over the past few decades, amounts of efforts have been made for the development of accurate and efficient docking tools, some of which are Autodock Vina [3], Autodock4 [4], GWOVina [5] and PSOVina [6]. In Autodock Vina (Vina), Markov Chain Monte Carlo (MCMC) is used as the global optimizer, which randomly mutates the most acceptable current solution to find the next worthier configuration. Broyden-Fletcher-Goldfard-Shanno (BFGS) plays the role of local search, which further exploits for more optimal solutions based on the results obtained by the global search of MCMC. In the other leading docking programs, there are similar executions with Vina, i.e., combining global and local algorithms. Autodock4 adopts a hybrid optimization method of global genetic algorithm (GA) and local Solis and Wets method. By using the grey wolf optimizer [7] (GWO) for global search and the BFGS for local search, GWOVina displays comparable docking performance with Vina but 2–7 times faster. The PSOVina [6] was developed by replacing the Monte Carlo method with particle swarm optimization (PSO) [8], achieving significant improvement for protein–ligand docking and comparable performance of virtual screening at five-to-six folds speedup state. Nevertheless, it has been observed from our experiments that the docking performance of PSOVina is considerably constrained by the fully parallel implementation.

The PSO method has been widely used for docking, since it has few parameters to adjust and its easy implementation. However, as Angeline pointed out [9], it is challenging for the canonical PSO to achieve a good balance between exploration (i.e. global search) and exploitation (i.e. local search) during the search process. To further improve the algorithmic search performance, a variety of strategies have been proposed, for examples, the adaptive parameters setting based on diversity control [10], social learning tactics [11], multi-swarm coevolution [12] and novel update equation for particles [13].

In this paper, based on the canonical PSO and random drift PSO (RDPSO), we propose a novel multi-swarm coevolution strategy with a master–slave model. In this model, there are one master swarm and multiple slave swarms, each slave devoting its best experience to the individual particle of the master swarm to promote the particle’s personal experience, and the master swarm passing back the particle’s personal experience to the corresponding slave swarm for further enhancing the slave’s exploration. It is verified by our experiments that the proposed multi-swarm model is more suitable for the complete parallelism than PSOVina, which makes the fully parallel implementation of the algorithm outperform Vina, PSOVina and GWOVina in terms of docking accuracy and screening capability whose quantity equals to Exhaustiveness parameter value.

## Methods

### Canonical and random drift PSO

The canonical PSO simulates the social behavior of bird flocking and fish schooling while searching for foods. The particle in PSO, like that fish or bird, represents a natural agent that possesses social behaviors. Examples of social behaviors include (1) improving the estimation accuracy of particle themselves to expected levels and (2) interacting with their neighborhood. For a PSO with $N$ particles and a D-dimensional search problem, the i-th particle at the n-th iteration has the velocity vector, the current position and the personal best (pbest) position with best fitness value obtained by the particle since initialization, represented as $V_{i,n} = (V_{i,n}^{1} ,V_{i,n}^{2} ,...,V_{i,n}^{D} )$, $X_{i,n} = (X_{i,n}^{1} ,X_{i,n}^{2} ,...,X_{i,n}^{D} )$ and $P_{i,n} = (P_{i,n}^{1} ,P_{i,n}^{2} ,...,P_{i,n}^{D} )$, respectively. In PSO, the ‘position’ item always means the position in the search space, and in this paper means the solution of the docking problem. Among all the pbest positions, the one with the best fitness value (the value of objective function, and in the docking task, namely, the scores of energy function) is known as the global best (gbest) position, denoted by $G_{n} = (G_{n}^{1} ,G_{n}^{2} ,...,G_{n}^{D} )$. The velocity and current position of the particle is updated as follows:

1 $$V_{i,n + 1}^{j} = \omega \cdot V_{i,n}^{j} + c_{1} \cdot r_{i,n}^{{}} \cdot (P_{i,n}^{j} - X_{i,n}^{j} ) + c_{2} \cdot R_{i,n}^{{}} \cdot (G_{n}^{j} - X_{i,n}^{j} )$$

2 $$X_{i,n + 1}^{j} = X_{i,n}^{j} + V_{i,n + 1}^{j}$$

where $\omega$, called inertia weight, is generally set to linearly decrease from 0.9 to 0.4 [14]. $r_{i,n}^{{}}$ and $R_{i,n}^{{}}$ are random numbers uniformly distributed on the interval of [0,1]. $c_{1}$ and $c_{2}$ are acceleration factors that are generally both set to be 2 as recommended by Shi and Eberhart [15].

Motivated by the trajectory analysis of PSO and the movement of electrons in a metal conductor placed in an external electric field, random drift particle swarm optimization, which uses a novel equation for updating the particle, has been proven to have better performance than the canonical PSO in most cases [13]. The update equations for RDPSO are expressed as:

3 $$V_{i,n + 1}^{j} = \alpha \left| {C_{n}^{j} - X_{i,n}^{j} } \right|\varphi_{i,n + 1}^{j} + \beta \left( {p_{i,n}^{j} - X_{i,n}^{j} } \right)$$

4 $$X_{i,n + 1}^{j} = X_{i,n}^{j} + V_{i,n + 1}^{j}$$

5 $$p_{i,n}^{j} = \gamma_{i,n}^{j} \cdot P_{i,n}^{j} + (1 - \gamma_{i,n}^{j} )G_{n}^{j}$$

where $C_{n}^{j}$ is the component in the $j$-th dimension of the mean best (mbest) position $C_{n} = (C_{n}^{1} ,C_{n}^{2} ,...,C_{n}^{D} )$ that is the average of all the pbest positions. $p_{i,n}^{j}$, defined by Eq. (5), is the component in the $j$-th dimension of the so-called local attractor of particle $i$. $\varphi_{i,n + 1}^{j}$ is a sequence of random numbers with standard normal distribution and $\gamma_{i,n}^{j}$ is a random number uniformly distributed between 0 and 1. $\alpha > 0$ and $\beta > 0$ are known as the thermal and drift coefficients, respectively. It is recommended that $\alpha$ decreases linearly from 0.9 to 0.3 and $\beta$ is constantly 1.45 [13].

### Multi-swarm cooperative model

**Fig. 1:**
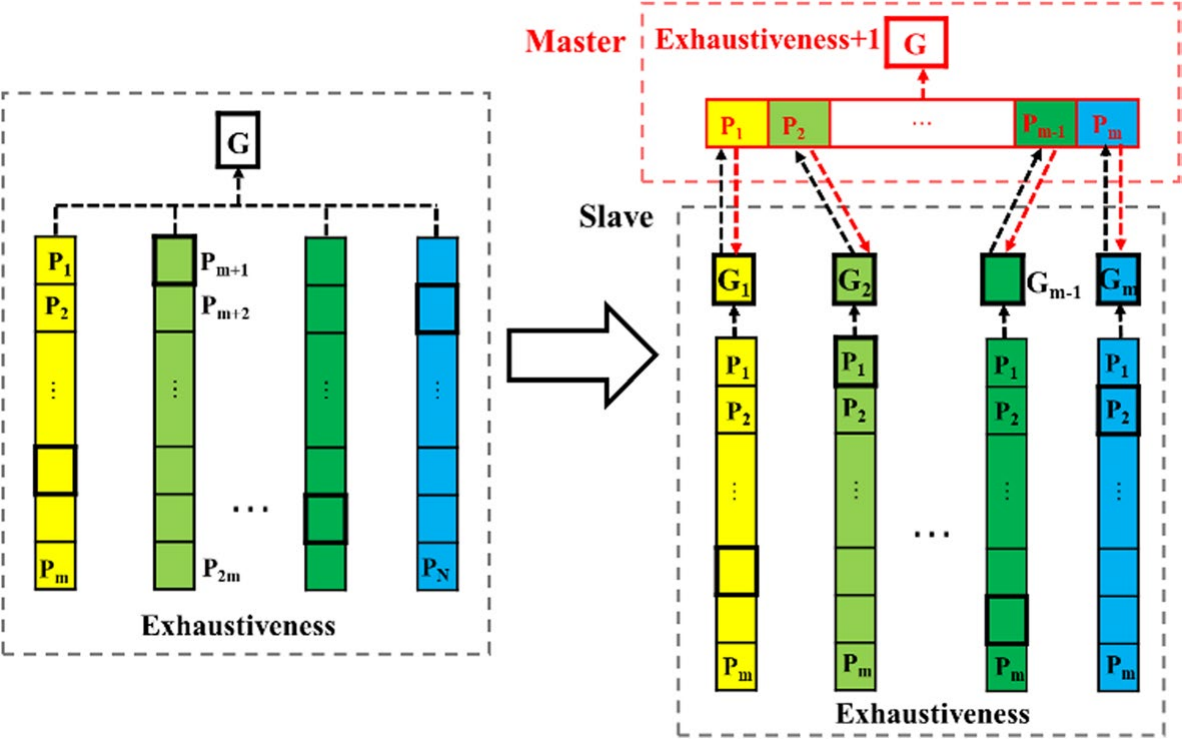
From the canonical model of PSOVina to our coevolution master–slave model. The arrows between master and slave subswarms represent the exchange between slave-subswarm’s gbest position and master-subswarm’s pbest position, and the rest arrows stand for the transferring from the best solution of corresponding swarms to the gbest position. More details are shown in the Support Information

In the canonical PSO, the search of particles is guided by self-cognitive and social experiences in order that they can move to promising regions. Such kind of mechanism is inspired by the social behavior of the mutual cooperation between individuals in a bird flock. However, in nature, besides the above individual cooperation within the same species, there is also another pervasive cooperation between different species, namely, the symbiosis cooperation, which can be divided into three types [16] (i.e. mutualism, commensalism and parasitism). Mutualism means that both species benefit from their interrelationship. Motivated by the mutualistic relationship, we propose a novel multi-swarm coevolution scenario in this paper. In our scenario, there are multiple slave swarms and one master swarm. The slave swarms are originated from the subswarms of PSOVina, but a little different. As illustrated in the left part of Fig. 1, the optimization population of PSOVina is composed of multiple same-sized subswarms, where the quantity of subswarm is equal to the value of exhaustiveness parameter. The number of particles in every subswarm is denoted by the num_particles parameter, and all the subswarms share the same gbest position. Unlike being subject to the only one gbest position in PSOVina, in our model shown as the right plot in Fig. 1, these slave subswarms possess their own gbest positions and evolve independently, which can be regard as original species. On the other hand, the master swarm is a new subswarm appended to our optimization population, and it essentially plays the role of another species and co-evolves with the original species.

The core of the mutualistic coevolution is the information exchange between the gbest experience of slave swarms and the pbest experience of the master swarm. During the co-evolution process, the slave swarms explores the whole solution space, while the master swarm exploits for solutions with higher precision. Each subswarm has access to slave-subswarm’s gbest and master-subswarm’s pbest information, and at the beginning of each iteration, there is a comparison between the values of slave-subswarm’s gbest fitness (gbest fitness of slave swarm) and master-subswarm’s pbest fitness (pbest fitness of master swarm) firstly executed. Referring to the exchanging arrows in Fig. 1, if the slave-subswarm’s gbest fitness is worse than the corresponding master-subswarm’s pbest fitness, the slave swarm substitutes slave-subswarm’s gbest position with master-subswarm’s pbest position (red arrows) to enhance the slave’s exploration and exploitation for promising solutions. Similarly, if slave-subswarm’s gbest fitness in the master swarm is better than the master-subswarm’s pbest fitness, slave-subswarm’s gbest position overrides master-subswarm’s pbest position (black arrows). That is, the master swarm makes use of the knowledge of slave swarms to improve its own exploitation ability. Overall, the mechanism of information exchange among coevolutionary subswarms makes the algorithm achieve an advanced balance between exploration and exploitation.

In our slave-master model, the slave swarms are all manipulated by the canonical PSO to supply the appropriate disturbance for algorithmic search. By utilizing the chaos-embedded PSO and random drift PSO for the guidance of master swarm, respectively, two docking tools are developed, called MPSOVina and MRDPSOVina, respectively.

### Algorithmic implementation details


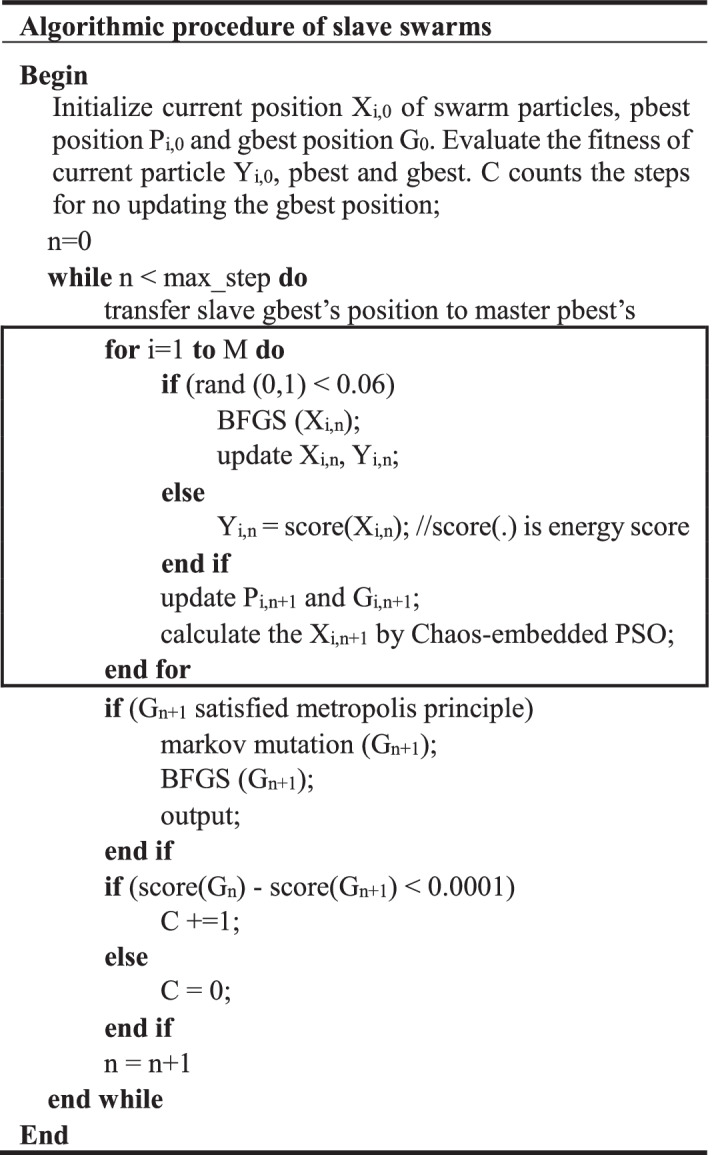



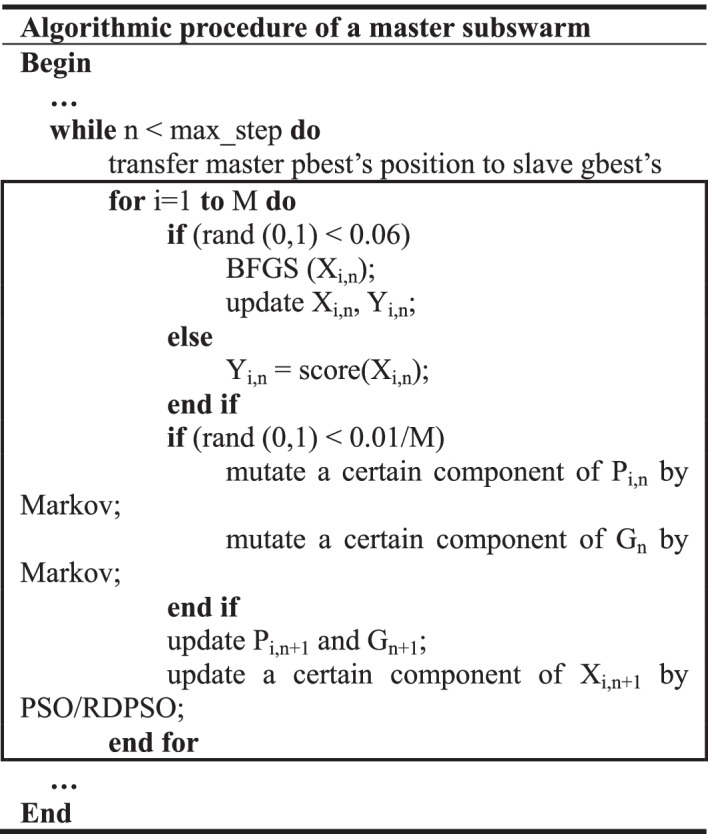


The previous section mentions the co-evolution between subswarms. In this section, the implementation details of single subswarm are illustrated. As the outlined above, the update procedure of a slave or master subswarm is marked by rectangle boxes. In each iteration, all the subswarm particles will utilize the global search method (PSO/RDPSO) to update their current positions. While for the local BFGS, each particle has only 6% possibility to employ it, since BFGS is very time-consuming. In slave swarms, the canonical PSO method changes all the components of particles’ current positions according to the Eqs. (1, 2). The current positions of the same particle between two adjacent iterations may be significantly different. The particles of slave swarms are able to be very randomly distributed in the entire solution space, and the slave swarms can effectively accomplish the exploration for solution space. In each iteration of a master swarm, the global search method only mutates one random component of a particle’s current position and thus the particle’s position can only be modified slightly. Using this manner, a particle in a master swarm, after absorbing the pbest experience of corresponding slave swarm, can readily develop better solutions around its pbest. Furthermore, the Markov mutation used in Vina is also employed in the master swarm, with the 0.001/M possibility to mutate one component of particles’ current position. This operation can generate more slight disturbance for pbest and gbest positions, which helps to obtain a better final solution.

Early experiments lead us to set the algorithmic parameters as follows. In canonical PSO, $\omega$ linearly decreases from 0.9 to 0.4, $c_{1}$, $c_{2}$, $r_{i,n}^{{}}$ and $R_{i,n}^{{}}$ are all set as same value in PSOVina. $c_{1}$ and $c_{2}$ are equal to 0.99. $r_{i,n}^{{}}$ and $R_{i,n}^{{}}$ are chaos-embedded random numbers between 0 and 1. The chaos-embedded PSO has exactly the same parameters with PSOVina. In RDPSO, $\alpha$ decreases linearly from 1.0 to 0.2, and $\beta$ is decreases linearly from 1.5 to 1.15.

### Datasets and experimental setups

To evaluate the performance of the proposed docking program in terms of pose prediction, the PDBbind core set v.2016 was utilized, which was systematically selected from the PDBbind refined set through a non-redundant sampling procedure and used as the dataset in CASF-2016 [17]. The core set collects 285 groups of protein–ligand complexes with high-precision resolution and provides the structural information of their separated proteins and ligands, all of which can be downloaded from http://www.pdbbind-cn.org. Moreover, we employed the cross-docking experiments on 8 protein-target families of CDK2, ESR1, F2, MAPK14, MMP8, MMP13, PDE4B and PDE5A from the Sutherland-crossdock-set, in which all the testing items are listed in the appendix reported by Dr. Jeff Sutherland [18]. Considering the relatively limited power of computation, 30 items in each of CDK2 (82 structures) and F2 (72 structures), as well as all the items available in the Protein Data Bank [19] for the rest families (< 30 structures) were selected and used for the crossing-docking executions.

The Database of Useful Decoys-Enhanced (DUD-E) was designed to benchmark the performance of virtual screening. In DUD-E, there are 22,886 active ligands and innumerable inactive ligands against 102 protein targets [20]. The inactive ligands, called decoys, have different topologies with active ligands but similar physicochemical properties that determine the protein–ligand interactions. Specifically, molecular weight, hydrogen bond acceptors and donors, and calculated log P dominate the van der Waals force, hydrogen-bonding and hydrophobic interaction, respectively. Therefore, it is challenging for docking tools to distinguish the real positive and positive-like ligands. In this work, since screening thousands of ligands against each target would expend large amounts of computational time, we only employed the four smallest subsets (ampc, cxcr4, cp3a4 and kif11) of DUD-E to compare the screening effectiveness of different docking tools.

Our docking experiments were implemented on the computational nodes of a cluster server with 240 processors of Intel(R) Xeon(R) E5–2620 based on a Centos 7.6.1810 Linux platform. All the docking simulations were performed according to the following three main steps, namely, preparation of the proteins and ligands, generation of the docking configuration files, and execution of the docking experiments. The input PDBQT files of protein and ligand were generated by prepare_protein4.py and prepare_ligand4.py, supplied by the AutoDock Tools. All the input parameters included the docking configuration files are identically default unless otherwise specified. In our docking experiments, all the specified parameters are illustrated as follows. The box size was 22.5 Å × 22.5 Å × 22.5 Å, the box center was the geometric center of the crystallized ligand, and the number of CPUs was set to different values to satisfy different experimental requirements.

### Performance metrics

Generally, the root mean squared deviation (RMSD) is used to estimate the prediction accuracy for binding poses of ligands, which reveals the overall difference of atomic positions (3D-coordinates of the ligand molecule) between prediction and co-crystallization. The RMSD calculation adopted in this paper is based on the heavy atoms of the ligand and excludes the consideration for structural symmetry. Only when RMSD is less than 2 Å, the predicted structure can be regarded as a successful docking. In this work, to reasonably assess the actual prediction accuracy, we conducted 10 groups of repetitive docking experiments and calculated the statistical results (e.g., mean RMSD, mean energy) of 10 docking repeats relative to the best-scored conformations for each testing item. The appraised error of the mean RMSD was obtained by 2000 rounds of bootstrapping [21]. The Wilcoxon signed-rank test undertaken at a 5% level of significance [22] was used to estimate the existence of significant difference between the RMSD-related results obtained by two docking programs.

The virtual screening is aimed to screen drug-like ligands from libraries of compounds. The evaluation for virtual screening performance is typically based on the list of compounds with binding energy ranked from low to high. A cutoff is set to categorize the compounds in the list as actives with lower energy and decoys with higher energy. In general, the larger number of truly active compounds, with lower energy than the cutoff, implies the higher screening capacity for a docking tool. Based on different cutoff settings, receiver operating characteristic (ROC) curves of docking tools can be created by plotting the false positive rate (FPR) against the true positive rate (TPR). In this paper, we utilized the area under the ROC curve (AUC) to assess the quality of virtual screening methods, whose value of 1.0 represents perfect classification ability, whereas 0.5 indicates random prediction [6]. Another performance metrics for virtual screening is the enrichment factor (EF) [6], expressed as follows:

6 $$EF_{x\% } = {{\frac{{top_{x\% } \, actives}}{{top_{x\% } \, ligands}}} \mathord{\left/ {\vphantom {{\frac{{top_{x\% } \, actives}}{{top_{x\% } \, ligands}}} {\frac{total \, actives}{{total \, ligands}}}}} \right. \kern-\nulldelimiterspace} {\frac{total \, actives}{{total \, ligands}}}}$$

where the $top_{x\% }$ ranked compounds are logically clustered as actives. The $EF_{x\% }$ is the concentration of the true activates among the $top_{x\% }$-scoring docking hits compared to their concentration throughout all the screened ligands. The larger value of $EF_{x\% }$ indicates better ability to find the true activates.

## Results and discussion

### Docking accuracy with different number of CPUs

In Vina and Vina-based programs, the actual parallel environment is dependent on the number of subswarms (specified by the exhaustiveness parameter), and the number of used CPUs (determined by the CPU parameter as well as the current available CPUs in computational machine). If the number of CPUs is larger than or equals to the number of subswarms (hereinafter named as adequate-CPU mode), each subswarm can be executed on one CPU, and thus all the subswarms can be run in parallel. On the contrary, if the number of used CPUs is not enough to support all the subswarms running simultaneously (the inadequate-CPU mode), part of subswarms firstly implements in parallel and then the remaining unevolved subswarms will orderly substitute the previously terminated subswarms.

**Fig. 2:**
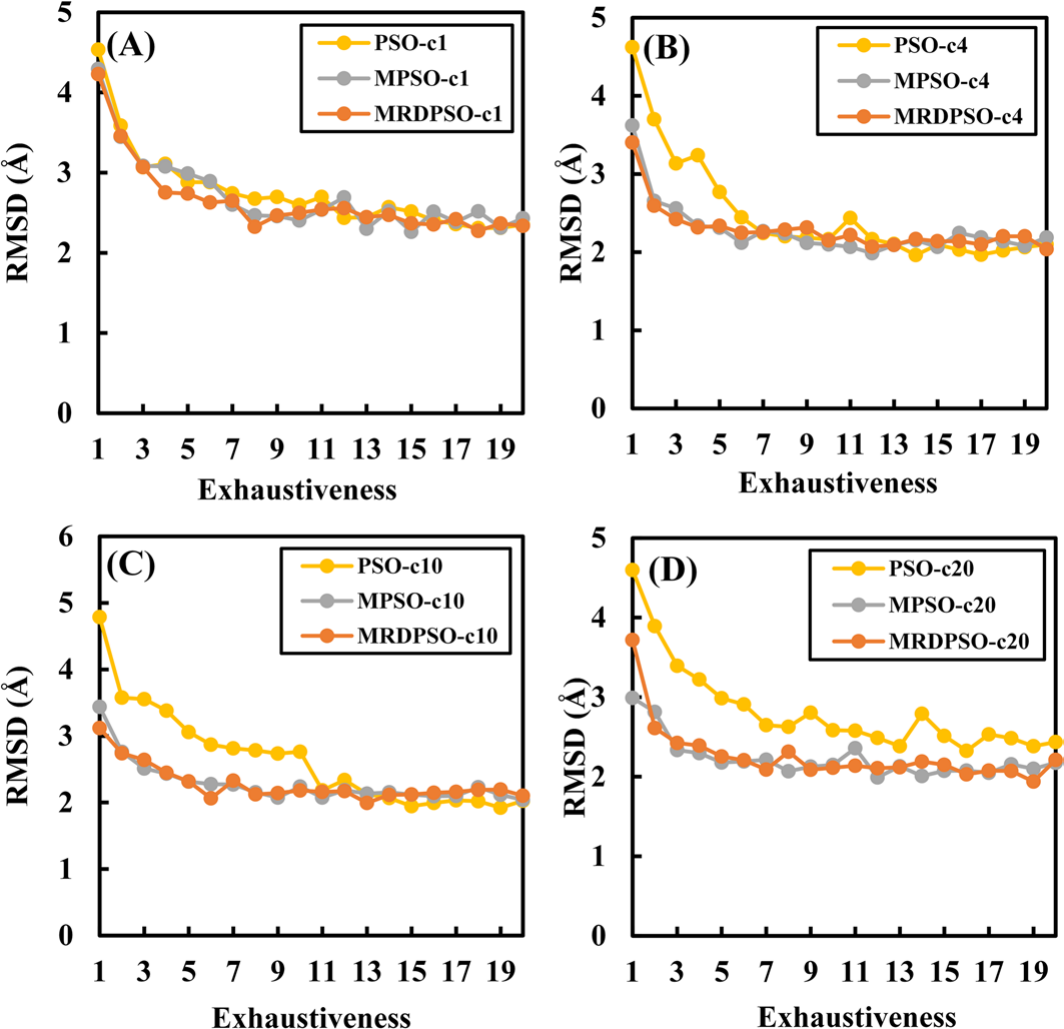
The docking comparison of PSOVina and multi-swarm programs with different number of CPUs. **A**, **B**, **C** and **D** sub-plots are for 1, 4, 10 and 20 CPUs, respectively

Figure 2 is derived from the docking experimental results obtained by PSOVina and multi-swarm programs in the execution environment of 1, 4, 10 and 20 CPUs (orderly correspond to A to D of Fig. 2). In Fig. 2A, PSOVina and our multi-swarm programs constantly ran in inadequate-CPU mode (no consideration of exhaustiveness = 1), and these docking programs exhibited the overall comparable docking performance. Moreover, it is obvious that the RMSD-results obtained by our proposed programs in Fig. 2A was worse than those in the other three subplots of Fig. 2, since there did not exist mutual beneficial cooperation between master swarm and slave swarms for the MPSOVina and MRDPSOVina in serial mode.

In Figs. 2B–D, for our proposed programs, there were not only individual particles’ chasing for gbest position within subswarms, but also mutual promotions between slave swarms and the master swarm, which significantly improved the docking performance. For the PSOVina in Fig. 2B–D, its performance lagged far behind that of the multi-swarm programs in adequate-CPU mode. The reason may be that the particles in all subswarms in PSOVina are simultaneously attracted by a single gbest and the whole swarm is very likely to be trapped in a local optimum. However, as shown in Fig. 2B, C, when the PSOVina was in inadequate-CPU mode, its docking results were much better than those in adequate-CPU mode, which may be attributed to the continued search by the bran-new subswarms, that is, the unevolved subswarms waiting for idle CPUs. Nevertheless, the results obtained by PSOVina in inadequate-CPU mode were still comparable to those obtained by our model.

Summarizing the above analysis, we can conclude that the docking performance of PSOVina excessively reckons on the number of CPUs, for the PSOVina only behaves well in inadequate-CPU mode. As a comparison, the MPSOVina and MRDPSOVina have outstanding docking performance in both inadequate- and adequate-CPU modes, indicating the good robustness of our proposed model.

### Docking performance for pose prediction

In this section, the default exhaustiveness value was set as 8, and most of the compared programs were run on 10 CPUs (adequate-CPU mode), only except the PSO-c4 and MRDPSO-c4 which are the PSOVina and the MRDPSOVina using 4 CPUs (inadequate-CPU mode), respectively. There were 12 × 8 = 96 wolves used in GWOVina, 8 × 8 = 64 particles in PSOVina, and 8 × 8 = 64 particles in the proposed multi-swarm programs.

Table 1 illustrates the overall accuracy, binding energy, P-value and running time obtained by all the compared docking programs. According to the results of mean RMSD value and mean success rate, MPSOVina and MRDPSOVina were able to achieve the most accurate docking prediction, followed by MRDPSO-c4 and PSO-c4, and finally Vina, PSOVina and GWOVina. This indicates that the master–slave mode can improve the pose prediction accuracy to a large extent. With regards to the binding energy, Vina obtained the lowest binding energy, followed by GWOVina and multi-swarm programs, and then PSOVina and PSO-c4. The proposed multi-swarm programs performed better than the classical Vina in terms of RMSD accuracy, but Vina generated the energy prediction of stronger binding, which might mainly be attributed to the poor effectiveness of scoring function. The calculated P-values reveal that MRDPSOVina had the significant difference with Vina, PSOVina, GWOVina and MRDPSO-c4, but was equivalent to PSO-c4 and MPSOVina. The PSO-c4 ran in inadequate-CPU mode and, according to the analysis in the previous section, it should obtain the comparable docking performance with MRDPSOVina. The reason why the docking results of MPSOVina and MRDPSOVina were similar may be that MPSOVina and MRDPSOVina both adopted the same search algorithms in the slave subswarms. With regards to the running time, which was estimated by docking experiments on the same computational node, MPSOVina and MRDPSOVina were consistently a little slower than PSOVina, but about 2 times and 9 times faster than GWOVina and Vina, respectively. Additionally, PSO-c4 was slower than MRDPSO-c4, and they both took more time than PSOVina and MRDPSOVina.

**Table 1:** Overall prediction accuracy, binding energy and running time comparison of Vina, PSOVina, GWOVina MPSOVina and MRDPSOVina

	Mean RMSD (Å)	Mean succ (%)	Mean energy ^a^	*P*-value ^b^	Mean time (s)
Vina	2.521 ± 0.328	64.35	− 8.680	2.73e-10	39.77
PSO-c4	2.283 ± 0.262	66.67	− 8.402	0.081	8.17
PSOVina	2.787 ± 0.274	59.47	− 8.274	3.99e-9	3.91
GWOVina	2.411 ± 0.317	65.72	− 8.671	2.87e-11	8.25
MPSOVina	2.133 ± 0.264	70.53	− 8.594	0.27	4.27
MRDPSO-c4	2.189 ± 0.272	69.75	− 8.572	0.0015	6.08
MRDPSOVina	2.056 ± 0.259	71.05	− 8.607	–	4.28

^a^The unite of mean binding energy is Kcal mol^−1^

^b^*P*-value is calculated by comparing two sets of mean RMSD results obtained by the MRDPSOVina and another docking method, respectively

**Fig. 3:**
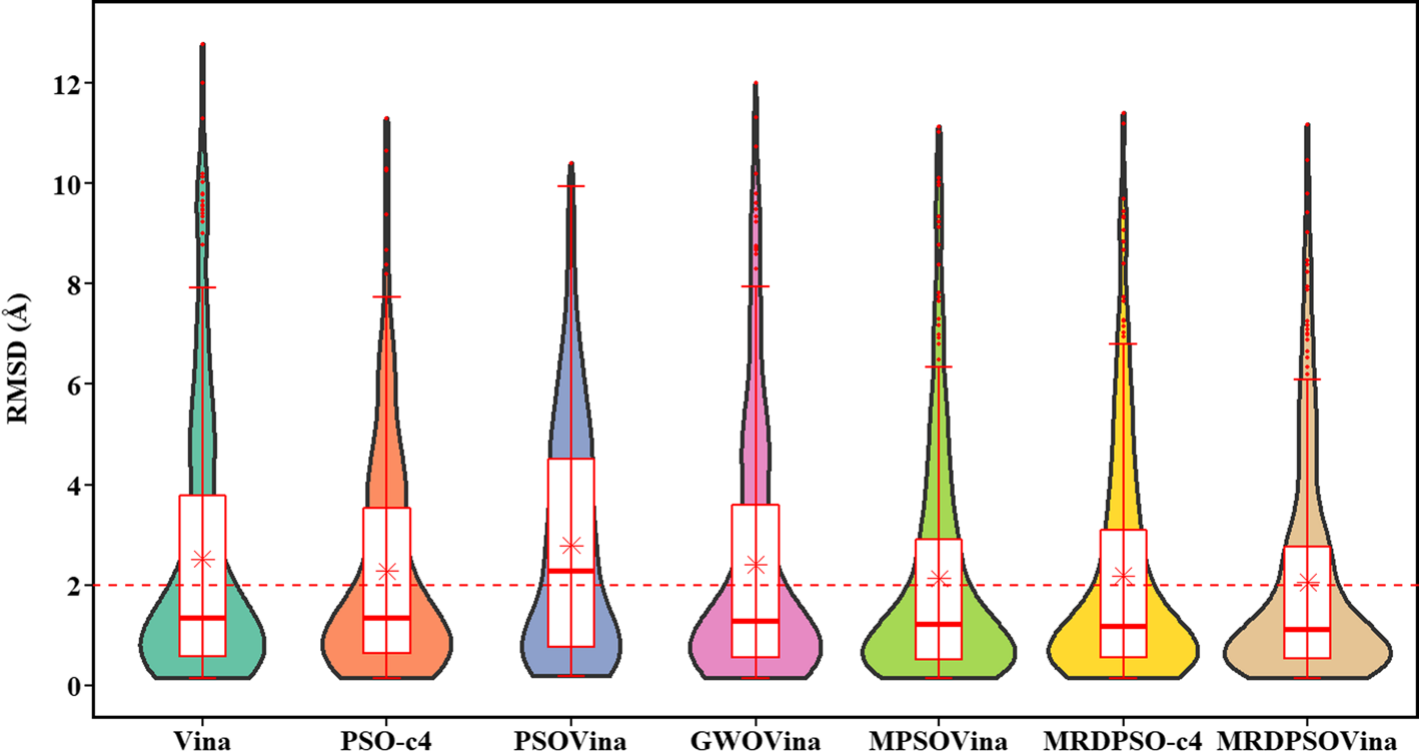
The box-and-whisker and violin plots for average RMSD comparison of all testing items obtained by Vina, GWOVina, PSOVina and multi-swarm programs. The dotted line is the dividing line of 2 Å. The red asterisk notations and the bold lines stand for the averages and medians of RMSD results of each docking program, respectively

The box-and-whisker and violin plots in Fig. 3 visualize the RMSD-result distribution of testing PDBbind dataset obtained by each docking program (all RMSD values are shown in Additional file 1: Table S2). Our proposed docking programs had lower box height and shorter whisker line than the other compared programs, which indicates that multi-swarm programs had the relatively more intensive RMSD distribution, that is, better docking stability than the others. In the violin plots, the width of a violin on a certain RMSD represents the occurrence frequency of the RMSD value in all RMSD results. The more violin areas mean more distribution of testing items. A horizontal line of 2 Å is created, and the violins give the distribution details of RMSD results. Above 2 Å line, there is more area of violin in PSO-c4 and PSOVina than the other docking tools, which represents more distribution of failed docking items for PSO-c4 and PSOVina than the other docking programs. On the other hand, it can be obviously observed that the RMSD results of our multi-swarm programs have more testing items distributed below the 2 Å line, which indicates the overall higher accuracy of these programs. Besides, the multi-swarm programs have the similar violin shapes, that is, comparable RMSD-result distribution and docking stability. Among the multi-swarm programs, MRDPSOVina has the most intensive RMSD distribution, especially below the 4 Å, which indicates the most stable and best docking effect.

The red asterisk notations and the bold lines in Fig. 3 stand for the averages and medians of RMSD results of each docking program, respectively. It is no doubt that the RMSD locations of red asterisk notations in Fig. 3 is consistent with the RMSD values in Table 1. In terms of the value of RMSD medians, only the PSOVina is beyond the 2 Å, so it docked over a half of the testing items as failed dockings and yielded the worst docking prediction among all the compared docking programs.

docking prediction among all the compared docking programs Additional file 1: Tables S1 and S2 in the Supplementary illustrate the mean energy and the mean RMSD obtained by each docking tool for the testing items with various number of torsions, respectively. Moreover, according to these statistical data, the polylines and the histograms of the Additional file 1: Figure S1 were generated, which implies the influence of different torsion number on docking program. From the polylines, it can be concluded that the PSO-c4 and PSOVina exhibited the weakest binding affinity across all the testing items of more than 7 torsions among all the compared docking programs, and the other docking programs yielded the similar binding energy throughout the torsion classifications. The histograms demonstrate that the mean RMSD of multi-swarm programs was lower than other compared docking programs in almost every class of the number of torsions. Hence, the multi-swarm programs can have better performance than the other compared approaches for dealing with the docking tasks no matter how many the numbers of torsions are, which also indicates the better docking stability of multi-swarm programs.

**Fig. 4:**
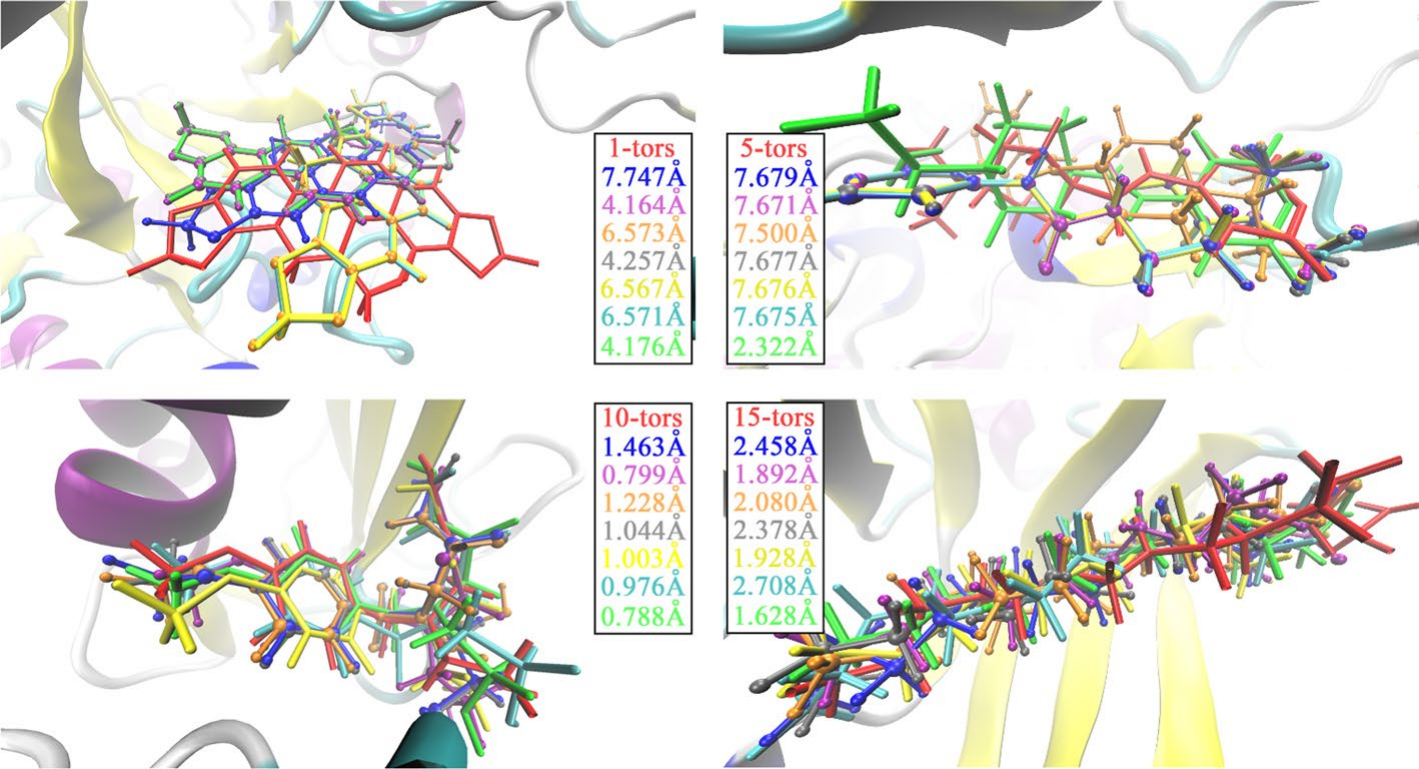
Visual comparison of the best-scored conformations obtained by different docking tools. Four testing items are selected and noted in the Additional file 1: Table S1. In the sub-plot for each item, there are three molecular visualizing models of ligands, i.e., bonds (crystallization), ball-and-stick (the compared results) and licorice (the results from our method). And each visual model displays with specific colors: the bonds with a red (crystallization) color, the ball-and-stick with blue (Vina), purple (PSO-c4), orange (PSOVina) and grey (GWOVina) colors and the licorice with yellow (MPSOVina), cyan (MRDPSO-c4) and green (MRDPSOVina) colors. The RMSD values of docking results, shown within the legend boxes, have the same color with visual ligand molecules

Furthermore, to make an intuitive performance comparison among different docking tools, we elaborately selected four ligands with 1, 5, 10 and 15 torsions, which come from the complexes of 3arv, 1q8t, 3nx7 and 3uew, respectively. The crystalline ligands and the best-scored conformations obtained by each docking tool are visualized in Fig. 4. Moreover, the corresponding RMSD values are listed in the legend. When it comes to the docking result of 1 torsion, PSO-c4, GWOVina and MRDPSOVina obtained the docking conformations of 4.164 Å, 4.257 Å and 4.176 Å, respectively, which are closer to the ligand crystallization than the docking results of the other methods. The MRDPSOVina yielded the second best docking conformation only behind the result of PSO-c4. For the docking results of 5 torsion ligands, the molecule geometry docked by MRDPSOVina obviously lays closer to crystalline structure than those by the other compared programs. With respect to the docking results for 10 and 15 torsions, MRDPSOVina generated the closest configurations to crystallized structures, and then PSO-c4 contributed the second best docking conformation, and the docking results of the other tools are all worse than those of MRPDPSOVina and PSO-c4. In summary, MRDPSOVina is a good choice of docking tools for docking different ligands with various torsion numbers.

**Fig. 5:**
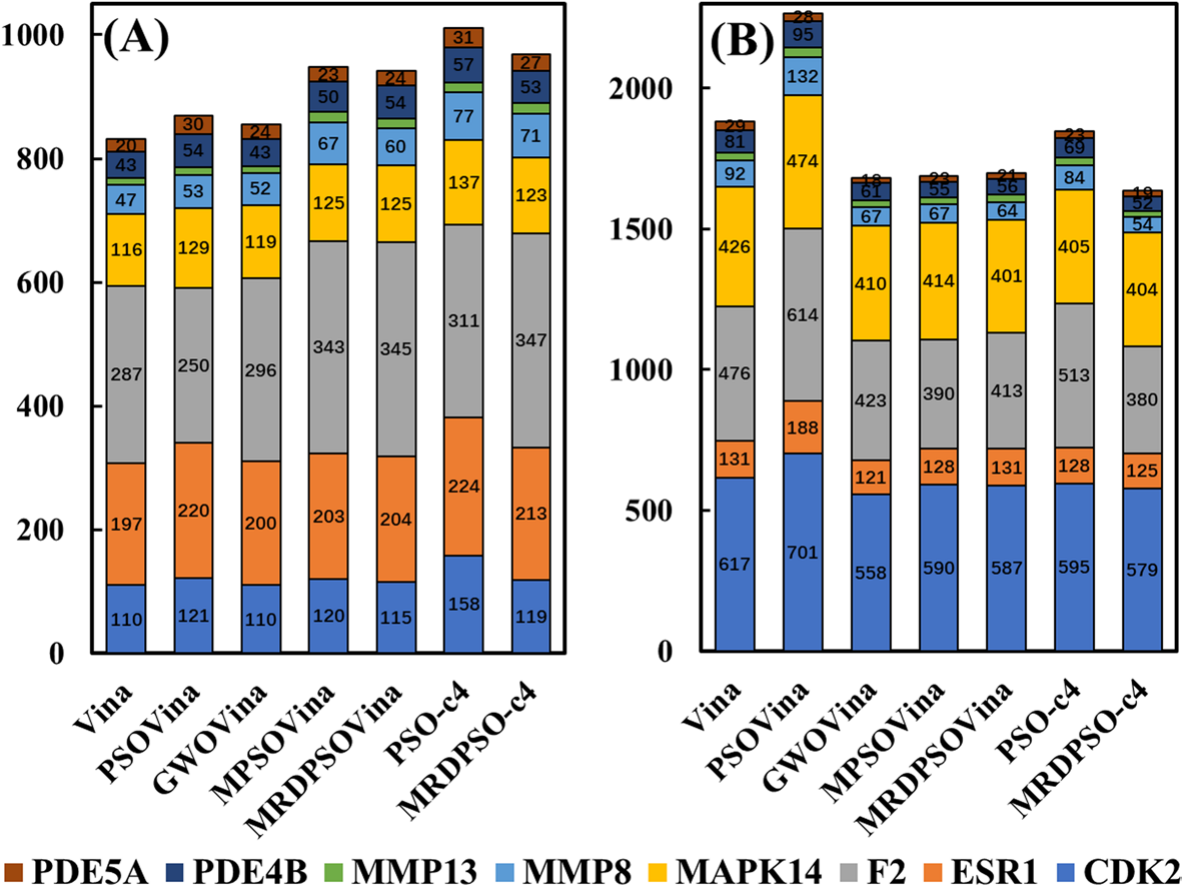
The statistical results of successful dockings and failed dockings for Vina-based programs. **A** illustrates the number of successful dockings for 8 family proteins. **B** shows that of failed dockings. The values on vertical bars are the number of successful or failed docking items

For the cross-docking target families, the experimental results are categorized as three clusters, namely successful docking, failed scoring and failed sampling, corresponding to three docking situations, respectively. The first cluster is that the obtained best-scored conformation is the correct pose with RMSD of less than 2 Å. The second one is that the correct pose docked to protein is not the best-scored one. The last one is that the RMSDs of the obtained configurations all exceed 2 Å. The Fig. 5 shows the numbers of successful dockings and the numbers of failed dockings for each protein family obtained by each compared docking program, and the cross-docking results of each protein target are illustrated by the corresponding statistical heat maps in the Additional file 1: Figure S2. It is obvious that MPSOVina and MRDPSOVina have more successful dockings and fewer failed dockings than Vina, PSOVina and GWOVina (GWOVina, MPSOVina ans MRDPSOVina have 1681, 1690, 1699 failed docking items in total, respectively), which indicates that multi-swarm models are more suitable for handling cross-docking tasks than the other docking programs in adequate-CPU mode. Moreover, MRDPSOVina is worse than MRDPSO-c4 in term of the cross-docking RMSD, and PSO-c4 is worse than PSOVina, which implies that the PSO-based docking methods in inadequate-CPU mode had higher cross-docking accuracy than those in adequate-CPU mode. PSO-c4 had the largest number of successful dockings among all the compared docking programs, but had more failed dockings than most of the compared docking programs. MRDPSO-c4 has the fewest failed dockings among all the compared docking programs, and has the second most successful dockings only behind the PSO-c4. Overall, MRDPSO-c4 was more effective than PSO-c4 in performing the cross-docking tasks.

### Performance comparison of virtual screening

**Fig. 6:**
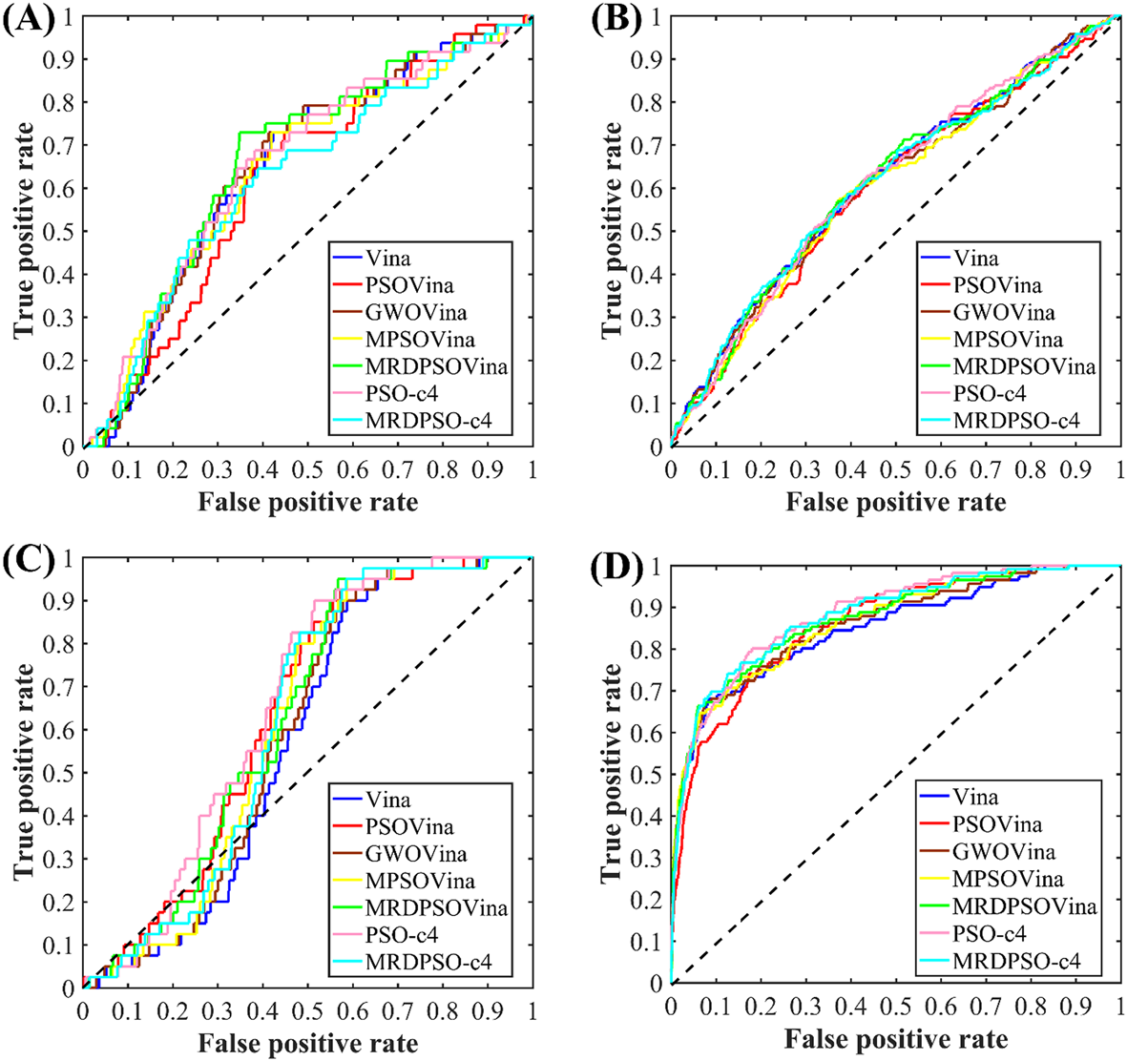
ROC curves of virtual screening four targets using all compared docking programs. **A** ROC curves for ampc. **B** ROC curves for cxcr4. **C** ROC curves for cp3a4. **D** ROC curves for kif11

To assess the virtual screening performance of the afore-mentioned docking methods, the ROC curves and the AUC values are utilized, and their results are presented in Fig. 6 and Table 2, respectively. In terms of the screening for ampc target, MRDPSOVina performed the best, and then PSO-c4 is the second best, followed by the other docking programs. For the cp3a4 protein target, all the docking programs exhibit the extremely similar ROC curves and have the comparable screening performance. With respective to the cxcr4 target, our proposed multi-swarm programs performed better than Vina and GWOVina, but not so well as PSOVina and PSO-c4. According to ROC and AUC results of kif11 target, PSO-c4 has the best screening performance, and our proposed programs has the second best, followed by Vina, PSOVina and GWOVina. In summary, MRDPSOVina and PSO-c4 can generate better screening results than the other docking programs. Referring to the latest report of PSOVina [6], we can find that PSOVina can generate better docking prediction than and comparable virtual screening to Vina, which coincides with our docking experimental results obtained by PSOVina in inadequate-CPU mode and screening results by PSOVina in adequate-CPU mode, respectively. When the docking tools run in inadequate-CPU mode, according to the AUC and ROC results of Table 2 and Fig. 6, MRDPSO-c4 performed worse than MRDPSOVina, but the performance of PSO-c4 turned out to be better than that of PSOVina and even better than that of MRDPSOVina.

**Table 2:** The AUC-ROC values of virtual screening four targets in DUD-E datasets

	Ampc	cp3a4	cxcr4	kif11	Mean	Time^a^
Vina	0.646	0.613	0.576	0.849	0.671	19.67
PSOVina	0.622	0.597	0.639	0.859	0.679	2.77
GWOVina	0.649	0.606	0.592	0.861	0.677	9.72
MPSOVina	0.644	0.598	0.613	0.865	0.680	4.13
MRDPSOVina	0.663	0.611	0.626	0.871	0.693	4.08
PSO-c4	0.653	0.611	0.655	0.883	0.701	6.31
MRDPSO-c4	0.626	0.610	0.620	0.879	0.684	6.32

^a^The unite of computational time is the second

The average time of docking per ligand by each program is shown in Table 2. It reveals that MRDPSOVina and MPSOVina had the second and the third fastest screening speed, respectively, and only slightly slower than PSOVina, but significantly faster than Vina and GWOVina. PSO-c4 and MRDPSO-c4 were almost in the unanimous speed and both were more computationally expensive than PSOVina and MRDPSOVina.

In the virtual screening, the focus is always put on the top ranked compounds that are generally regarded as positive compounds and worth to further explore their drug-like properties. In this paper, the top_1%_, top_2%_ and top_5%_ ranked compounds are logically clustered as actives, respectively, and three corresponding enrichment factors of EF_1%_, EF_2%_ and EF_5%_ are reported in Table 3. Among all the compared programs, MPSOVina had the best screening performance in terms of EF_1%_ and EF_2%_, followed by MRDPSOVina. As for the results of EF_5%_, the MPSOVina, MRDPSOVina, PSO-c4 and GWOVina exhibited the comparable performance. Typically, the ligands at more top ranked locations are consider as more worthy candidates to explore their pharmaceutical property, and thus EF_1%_ and EF_2%_ are more representative than EF_5%_ for the evaluation of virtual screening. Compared with other docking programs, the MPSOVina accomplished the best, and the MRDPSOVina had an outstanding enrichment effect for drug-like candidates. These superior results mainly benefited from our proposed multi-swarm model.

**Table 3:** The EF values of virtual screening four targets in DUD-E dataset

	EF (%)	ampc	cp3a4	cxcr4	kif11	Mean
Vina	1	0	3.593	0	24.14	6.933
	2	0	2.695	0	18.53	5.307
	5	0	2.156	0.5	10.69	3.337
PSO-c4	1	0	3.593	2.5	24.14	7.558
	2	1.042	2.096	1.25	17.67	5.515
	5	0.833	1.796	0.5	11.03	3.541
PSOVina	1	0	1.796	2.5	19.83	6.031
	2	1.042	1.796	1.25	12.93	4.255
	5	0.417	1.796	0.5	9.31	3.006
GWOVina	1	0	3.593	0	25.86	7.364
	2	0	2.695	0	18.97	5.415
	5	0.417	2.275	1	10.69	3.596
MPSOVina	1	0	4.192	0	28.45	8.16
	2	0	2.395	1.25	18.97	5.653
	5	0.833	2.036	0.5	10.86	3.558
MRDPSO-c4	1	0	2.994	0	24.14	6.783
	2	0	2.395	1.25	16.81	5.114
	5	0.833	1.916	0.5	10.52	3.442
MRDPSOVina	1	0	3.593	0	27.59	7.795
	2	0	2.695	1.25	18.53	5.62
	5	0.417	2.156	0.5	11.03	3.527

## Conclusions

In this work, based on PSO and RDPSO, we proposed a novel multi-swarm coevolution scenario with a master–slave model, and applied it to the docking algorithm. In our coevolutionary model, there are self-independent evolutions of subswarms and information exchanges between slave gbest positions and master pbest positions. Our docking experimental results demonstrated that multi-swarm programs are more robust for the parallel execution than PSOVina, and have better stable performance of docking prediction and virtual screening than Vina and GWOVina. The better parallel robustness attributes to the independent evolution of subswarm, and the information exchanges, bringing the improvement of algorithmic explorations and exploitations, contribute to the better docking effects.

Moreover, in our experiments, MRDPSOVina, among our multi-swarm programs, served as an efficient docking tool and exhibited the best docking performance. On the one hand, MRDPSOVina has the best prediction accuracy of ligand pose, the most concentrated RMSD distribution, and about two-fold and nine-fold faster speed than GWOVina and Vina, respectively. On the other hand, MRDPSOVina can harvest the AUC prediction only inferior to PSO-c4, and an outstanding performance in terms of enrichment factors. These indicate that fully parallel implementation is essential to the efficiency improvement of docking programs, and the RDPSO is a remarkable method for docking optimization problems.

## Supplementary Information

**Additional file1**: the RMSD and energy results of all test cases in PDBbind core set and the cross-docking results of eight protein families in Sutherland-crossdock-set. (PDF 1099 kb)

## Data Availability

The PDBbind dataset can be accessed on http://www.pdbbind-cn.org/casf.aspa. The details of Sutherland-crossdock-set are shown on https://pubs.acs.org/doi/suppl/10.1021/ci700253h/suppl_file/ci700253h-file001.pdf and the corresponding crystallized complexes are accessible on the Protein Data Bank repository, https://www.rcsb.org/.
