# Supplementary material for: Parallel multi-swarm cooperative particle swarm optimization for protein–ligand docking and virtual screening
Source: BMC Bioinformatics. 2022 May 30;23:201. doi: 10.1186/s12859-022-04711-0 (PMC9150318; doi:10.1186/s12859-022-04711-0)
Supplement: Supplementary file 1 — Additional file1: the RMSD and energy results of all test cases in PDBbind core set and the cross-docking results of eight protein families in Sutherland-crossdock-set. (PDF 1099 kb) [file 12859_2022_4711_MOESM1_ESM.pdf]

Supplementary material for

Parallel Multi-swarm Cooperative Particle Swarm Optimization for protein-ligand docking and virtual screening

Chao Li[1], Jinxing Li[1], Jun Sun*[1], Li Mao[1], Vasile Palade[2] , Bilal Ahmad[1]

[1] Department of Computer Science and Technology, Jiangnan University, No.1800, Lihu Avenue, Wuxi, Jiangsu, 214122, People's Republic of China

[2] Center for Computational Science and Mathematical Modeling, Coventry University, Priory Street, Coventry, CV15FB, UK

More Details for Figure 1

The original optimization population in PSO of PSOVina, shown in the left black dotted box, consists of multiple subswarms of N particles. The quantity of subswarms is equal to Exhaustiveness value, and different subswarms are expressed as differently colored columns. The right part of figure displays our optimization population, which is composed of slave subswarms of N particles and a master subswarm with m particles. The master subswarm is the (Exhautiveness+1)-th subswarm that is newly added and shown as red color.

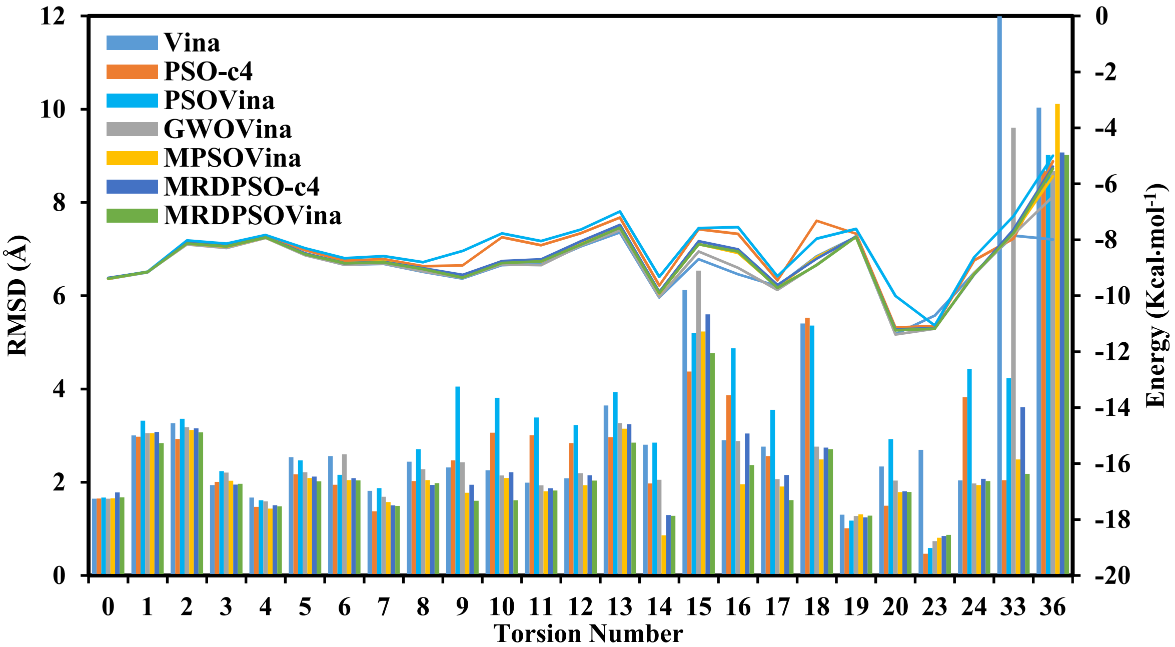

Figure S1. The average RMSD and energy comparison of molecules with different torsions.

Table S1. The scored-energy of 285 test cases obtained by all compared docking programs

		Scored-Energy (Kcal•mol-1)						
**PDB**	**ntor**	**Vina**	**PSO-c4**	**PSOVina**	**GWOVina**	**MPSOVina**	**MRDPSO-c4**	**MRDPSOVina**
**3jya**	**0**	-8.29	-8.26952	-8.23285	-8.25906	-8.26704	-8.27321	-8.25564
**3udh**	**0**	-7.6	-7.60711	-7.60686	-7.60628	-7.60601	-7.5183	-7.60686
**4kzu**	**0**	-12.3	-12.2985	-12.3011	-12.2987	-12.3	-12.2989	-12.3003
**Average**		**-9.39667**	**-9.39171**	**-9.38027**	**-9.38801**	**-9.39103**	**-9.36347**	**-9.38758**

**1bcu**	**1**	-7.5	-7.46477	-7.46625	-7.4637	-7.4639	-7.46458	-7.46436
**1gpn**	**1**	-10.88	-10.8516	-10.8513	-10.8513	-10.8495	-10.8518	-10.8516
**2xys**	**1**	-11.2	-11.1769	-11.1794	-11.173	-11.1765	-11.1767	-11.1711
**3arv**	**1**	-11	-10.9313	-10.9515	-10.964	-10.9491	-10.9564	-10.9569
**3rsx**	**1**	-7.8	-7.83536	-7.74313	-7.83371	-7.81408	-7.83302	-7.83496
**4ddk**	**1**	-6.61	-6.63366	-6.63074	-6.63062	-6.63414	-6.63377	-6.63648
**Average**		**-9.165**	**-9.14893**	**-9.13705**	**-9.15273**	**-9.14785**	**-9.1527**	**-9.15256**
**1c5z**	**2**	-5.8	-5.76589	-5.76628	-5.76831	-5.76748	-5.76579	-5.7665
**1o5b**	**2**	-6.6	-6.55485	-6.55505	-6.56257	-6.56305	-6.56358	-6.532
**1r5y**	**2**	-7.4	-7.22581	-6.97486	-7.41181	-7.41542	-7.41358	-7.28528
**1s38**	**2**	-7.8	-7.8254	-7.42735	-7.81165	-7.80843	-7.62361	-7.80763
**2iwx**	**2**	-9	-9.01123	-9.01122	-9.01161	-9.01155	-9.01125	-9.01126
**2weg**	**2**	-6.2	-6.13675	-5.83276	-6.18656	-6.13358	-6.0295	-6.10743
**2yki**	**2**	-15.56	-16.5619	-16.4941	-16.5621	-16.563	-16.5466	-16.5575
**3ary**	**2**	-7.72	-7.49516	-7.36808	-7.70964	-7.62023	-7.59803	-7.64732
**3g2z**	**2**	-6.87	-6.19426	-6.22541	-6.85998	-6.59252	-6.66449	-6.56383
**3gv9**	**2**	-5.3	-4.90624	-4.8928	-5.34351	-5.2288	-5.13603	-5.29462
**3kr8**	**2**	-12	-11.9951	-11.9959	-11.9985	-11.998	-11.9987	-11.9988
**3pxf**	**2**	-10.3	-10.2837	-10.1745	-10.2039	-10.175	-10.1976	-10.2746
**3twp**	**2**	-5.5	-5.39845	-5.36154	-5.47726	-5.42926	-5.39513	-5.4488
**3u5j**	**2**	-8.7	-8.70849	-8.70784	-8.70985	-8.708	-8.57734	-8.70706
**3zt2**	**2**	-9.73	-9.73857	-9.5749	-9.7661	-9.75194	-9.75348	-9.73996
**4f09**	**2**	-8.1	-8.13877	-8.13727	-8.13669	-8.13856	-8.13868	-8.13981
**4gfm**	**2**	-7.81	-7.83044	-7.82084	-7.83062	-7.83549	-7.83215	-7.83453
**4jsz**	**2**	-5.28	-5.17107	-5.15606	-5.25978	-5.12507	-5.19935	-5.14348
**4k77**	**2**	-8.14	-8.13781	-8.05314	-8.21151	-8.16446	-8.14816	-8.1801
**4kzq**	**2**	-11.11	-11.1449	-11.1445	-11.1409	-11.1432	-11.1319	-11.1411
**4mme**	**2**	-10.3	-10.2632	-10.2651	-10.2629	-10.2674	-10.2644	-10.2654
**4owm**	**2**	-5.4	-5.3701	-5.34065	-5.38479	-5.38417	-5.37965	-5.36773
**4u4s**	**2**	-6.3	-6.22296	-6.21874	-6.28802	-6.28241	-6.28347	-6.24
**Average**		**-8.12696**	**-8.09048**	**-8.02169**	**-8.1695**	**-8.13509**	**-8.11532**	**-8.13282**
**1e66**	**3**	-12.1	-12.121	-12.1181	-12.1179	-12.1185	-12.1176	-12.1187
**1gpk**	**3**	-9.9	-9.92557	-9.92391	-9.924	-9.92458	-9.92432	-9.924
**1qkt**	**3**	-10.4	-10.353	-10.353	-10.3533	-10.3525	-10.3519	-10.353
**1uto**	**3**	-4.6	-4.54623	-4.49948	-4.57161	-4.56247	-4.56223	-4.5736
**1ydr**	**3**	-8.19	-8.15067	-8.11365	-8.15346	-8.1543	-8.15287	-8.1519
**2pog**	**3**	-10.3	-10.2654	-10.2662	-10.2655	-10.2658	-10.265	-10.2651
**2wer**	**3**	-8.8	-8.7669	-8.7669	-8.7692	-8.7669	-8.76702	-8.76695
**3acw**	**3**	-10.95	-10.9425	-10.9472	-10.9491	-10.9403	-10.9446	-10.9504
**3ao4**	**3**	-7.45	-7.30944	-7.13426	-7.44897	-7.43175	-7.43742	-7.36924
**3bgz**	**3**	-10.9	-10.9225	-10.9203	-10.9218	-10.9206	-10.9234	-10.9228
**3g31**	**3**	-5	-4.71843	-4.73352	-4.96841	-4.9662	-4.86076	-4.91351
**3gy4**	**3**	-6.13	-6.05207	-6.05336	-6.28246	-6.07903	-6.05776	-6.0551
**3kgp**	**3**	-5.7	-5.74779	-5.73657	-5.75245	-5.74974	-5.73044	-5.72357
**3pyy**	**3**	-9.1	-9.12769	-9.00722	-9.13391	-9.01559	-9.12447	-9.1197
**3wtj**	**3**	-7	-6.95947	-6.94649	-7.00029	-6.98699	-6.85266	-6.974
**4cr9**	**3**	-6.7	-6.70902	-6.67282	-6.69373	-6.71108	-6.66646	-6.70688
**4de1**	**3**	-8.38	-7.68563	-7.26008	-8.39775	-7.89639	-8.01829	-7.98866
**4de3**	**3**	-8.51	-8.58176	-8.1759	-8.48106	-8.61127	-8.41018	-8.43118
**4e6q**	**3**	-9.8	-9.76467	-9.75671	-9.80233	-9.79333	-9.78109	-9.78558
**4ih5**	**3**	-6.8	-6.43544	-6.47873	-6.74163	-6.55033	-6.53598	-6.60411

**4j21**	**3**	-11.6	-11.5588	-11.5593	-11.5591	-11.5585	-11.5573	-11.5591
**4llx**	**3**	-4.99	-5.07883	-5.06882	-5.16545	-5.10303	-5.08622	-5.09951
**4m0y**	**3**	-9.3	-9.0704	-8.7842	-9.33128	-9.32491	-9.23531	-9.15163
**5c28**	**3**	-6.2	-6.00667	-6.02266	-6.18457	-6.1842	-6.13085	-6.18666
**Average**		**-8.28333**	**-8.19999**	**-8.13747**	**-8.29038**	**-8.24868**	**-8.22892**	**-8.23728**
**1q8u**	**4**	-8.4	-8.36952	-8.36944	-8.36887	-8.36832	-8.37007	-8.3687
**1syi**	**4**	-9.1	-9.12155	-9.12203	-9.12595	-9.12655	-9.12263	-9.1268
**2al5**	**4**	-8.88	-8.9164	-8.91663	-8.9145	-8.91715	-8.91995	-8.91633
**2cbv**	**4**	-7.13	-7.04962	-7.13332	-7.20412	-7.20493	-7.13252	-7.1341
**2hb1**	**4**	-6.39	-6.04744	-5.51219	-6.35967	-6.28005	-6.35918	-6.3261
**2j7h**	**4**	-6.2	-6.19865	-6.12374	-6.20177	-6.20203	-6.20106	-6.19856
**2v00**	**4**	-7.8	-7.77035	-7.53086	-7.78857	-7.78033	-7.78299	-7.63238
**2wnc**	**4**	-8.3	-8.31595	-8.24443	-8.29199	-8.29072	-8.29627	-8.31868
**2wtv**	**4**	-11.6	-11.6002	-11.5914	-11.6086	-11.5952	-11.5981	-11.6015
**2ymd**	**4**	-7.28	-7.447	-7.44726	-7.44487	-7.44664	-7.44813	-7.43972
**3b27**	**4**	-6.9	-6.92359	-6.83008	-6.92074	-6.92377	-6.9218	-6.9212
**3dx1**	**4**	-6.8	-6.46587	-6.58486	-6.76469	-6.76519	-6.67473	-6.76539
**3f3a**	**4**	-8.09	-8.05785	-7.97932	-8.06144	-8.06771	-8.05858	-8.06021
**3f3c**	**4**	-7.9	-7.93822	-7.93821	-7.94406	-7.93732	-7.94015	-7.93776
**3g0w**	**4**	-10.9	-10.8591	-10.8609	-10.8606	-10.8601	-10.8627	-10.8622
**3gr2**	**4**	-5.9	-5.6737	-5.68184	-5.88767	-5.8108	-5.78098	-5.73078
**3lka**	**4**	-6.7	-6.62651	-6.65382	-6.72338	-6.74076	-6.7364	-6.69093
**3n7a**	**4**	-7.52	-7.56905	-7.5723	-7.5655	-7.5887	-7.56554	-7.57401
**3qqs**	**4**	-8.66	-8.58994	-8.59781	-8.66239	-8.591	-8.6465	-8.57492
**3rr4**	**4**	-8.1	-7.9968	-7.64063	-8.07222	-8.09455	-8.10659	-8.10022
**3u8k**	**4**	-7.1	-7.06323	-7.05988	-7.06115	-7.06479	-7.06662	-7.06609
**3u8n**	**4**	-7.41	-7.41107	-7.44145	-7.43965	-7.44079	-7.44139	-7.43621
**4abg**	**4**	-5.79	-5.5877	-5.51668	-5.76142	-5.6679	-5.6694	-5.66892
**4ddh**	**4**	-7.3	-7.15163	-7.05609	-7.27309	-7.23213	-7.22529	-7.22062
**4dli**	**4**	-10	-10.0343	-10.0347	-10.0355	-10.0372	-10.0376	-10.0332
**4hge**	**4**	-8.7	-8.91181	-8.73777	-8.68858	-8.68988	-8.70169	-8.83495
**4ivb**	**4**	-9.7	-9.73716	-9.73703	-9.73767	-9.73714	-9.73724	-9.73744
**4j28**	**4**	-7.3	-7.29096	-7.2609	-7.28704	-7.29021	-7.21993	-7.29103
**Average**		**-7.92321**	**-7.88304**	**-7.8277**	**-7.93056**	**-7.91971**	**-7.91514**	**-7.91318**
**1nc3**	**5**	-9.8	-9.78649	-9.78955	-9.78907	-9.78733	-9.78737	-9.78551
**1o3f**	**5**	-8.89	-8.64203	-7.94988	-8.89972	-8.88383	-8.77963	-8.86436
**1oyt**	**5**	-10.92	-10.9491	-10.9275	-10.9442	-10.9499	-10.9469	-10.9484
**1p1n**	**5**	-8.1	-8.10344	-8.09713	-8.10207	-8.09556	-8.08923	-8.09321
**1p1q**	**5**	-7.49	-7.43261	-7.3891	-7.49853	-7.48918	-7.47788	-7.47491
**1ps3**	**5**	-8.2	-7.99013	-8.19479	-8.19505	-8.19479	-8.19553	-8.19695
**1q8t**	**5**	-7.8	-7.77236	-7.71928	-7.81643	-7.77798	-7.78384	-7.79713
**2r9w**	**5**	-10.24	-9.35382	-8.7688	-10.2573	-9.82737	-9.6752	-9.64525
**2wn9**	**5**	-8.3	-7.42704	-7.44785	-8.33629	-7.7272	-8.03733	-8.03596
**2xj7**	**5**	-8.61	-8.64526	-8.64629	-8.64618	-8.64578	-8.64752	-8.64713
**3d4z**	**5**	-7.15	-6.28014	-6.5027	-7.14562	-7.16821	-6.85787	-7.14621
**3d6q**	**5**	-6.6	-6.57576	-6.1232	-6.53028	-6.58775	-6.45726	-6.63585
**3dd0**	**5**	-6.3	-6.2298	-5.92896	-6.28692	-6.28446	-6.27871	-6.27931
**3fcq**	**5**	-5.5	-5.54807	-5.47024	-5.63951	-5.62015	-5.61545	-5.60651
**3fur**	**5**	-11.31	-11.5196	-11.6039	-11.6574	-11.6649	-11.6597	-11.6597
**3gbb**	**5**	-9.43	-9.45707	-9.46941	-9.4494	-9.44801	-9.42094	-9.46752
**3jvr**	**5**	-7.69	-7.20982	-6.68433	-7.69953	-7.69929	-7.57968	-7.6832

**3qgy**	**5**	-9.51	-9.40725	-9.27442	-9.54179	-9.51511	-9.47585	-9.52262
**3rlr**	**5**	-8.9	-8.58633	-8.74304	-8.87756	-8.87481	-8.75266	-8.867
**3ryj**	**5**	-7.3	-7.25446	-7.18163	-7.2816	-7.2313	-7.27255	-7.24871
**3uuo**	**5**	-7.71	-7.74798	-7.74179	-7.74851	-7.74582	-7.73714	-7.74563
**4e5w**	**5**	-10.1	-9.80644	-9.76004	-10.0812	-10.0838	-10.0808	-10.0793
**4f9w**	**5**	-10	-9.8464	-9.774	-9.97033	-9.9744	-9.87054	-9.97852
**4gkm**	**5**	-8.4	-8.40208	-8.5217	-8.40451	-8.46939	-8.43613	-8.4588
**4ih7**	**5**	-7	-6.97604	-6.98075	-6.97559	-6.97373	-6.97704	-6.97334
**4ivc**	**5**	-9.7	-9.71705	-9.67261	-9.72137	-9.71971	-9.71649	-9.71777
**4k18**	**5**	-10.8	-10.5182	-10.5216	-10.8264	-10.8281	-10.7022	-10.7513
**4kz6**	**5**	-5.7	-5.60171	-5.36857	-5.74245	-5.63413	-5.65212	-5.68613
**4m0z**	**5**	-10.9	-10.8914	-10.8867	-10.8836	-10.8853	-10.8847	-10.8899
**4mgd**	**5**	-8.61	-8.59521	-8.6026	-8.63175	-8.59509	-8.6056	-8.60029
**4pcs**	**5**	-7.2	-7.18022	-7.1805	-7.20298	-7.17984	-7.18893	-7.18418
**4qac**	**5**	-11.27	-11.257	-11.2583	-11.255	-11.2549	-11.2515	-11.258
**5aba**	**5**	-6	-5.50487	-5.63886	-6.05027	-5.90943	-6.05498	-6.03658
**Average**		**-8.52818**	**-8.37016**	**-8.29758**	**-8.54813**	**-8.50686**	**-8.48331**	**-8.5141**
**1owh**	**6**	-14	-13.9689	-13.9809	-13.9819	-13.9716	-8.20316	-13.9798
**1pxn**	**6**	-8.4	-8.28315	-8.15497	-8.39977	-8.35793	-8.84503	-8.29865
**1w4o**	**6**	-8.8	-8.82301	-8.79289	-8.84449	-8.83753	-6.68985	-8.84237
**2brb**	**6**	-6.7	-6.66241	-6.64869	-6.66858	-6.68248	-8.12552	-6.68962
**2c3i**	**6**	-8.19	-8.1316	-8.04439	-8.19753	-8.0966	-8.90511	-8.14736
**2j78**	**6**	-9	-8.74204	-8.74661	-9.03127	-8.91014	-6.60524	-8.92039
**2p15**	**6**	-6.6	-6.43966	-6.27502	-6.58394	-6.58713	-13.8019	-6.61557
**2qe4**	**6**	-13.8	-13.8078	-13.7999	-13.8046	-13.8003	-9.74897	-13.8044
**2xnb**	**6**	-9.73	-9.75173	-9.75317	-9.7535	-9.75014	-10.3112	-9.75153
**3dx2**	**6**	-10.36	-10.1086	-9.91056	-10.3644	-10.3269	-6.7059	-10.3393
**3dxg**	**6**	-6.7	-6.26076	-5.84109	-6.70755	-6.61835	-6.3781	-6.59523
**3e93**	**6**	-6.4	-6.20367	-6.11646	-6.41952	-6.34732	-13.9824	-6.36939
**3ebp**	**6**	-9.7	-9.67356	-9.53204	-9.67169	-9.68303	-9.68086	-9.67528
**3f3d**	**6**	-5.8	-5.72966	-5.75522	-5.77437	-5.77832	-5.77343	-5.77542
**3f3e**	**6**	-6.3	-6.28565	-6.27669	-6.30987	-6.28849	-6.31853	-6.29449
**3gc5**	**6**	-9.6	-9.20643	-9.34282	-9.56467	-9.51414	-9.39069	-9.56615
**3k5v**	**6**	-9.9	-9.59632	-9.45976	-9.92882	-9.72818	-9.74431	-9.74361
**3l7b**	**6**	-9	-8.97908	-8.98119	-8.97765	-8.97889	-8.97916	-8.9785
**3r88**	**6**	-5.74	-5.53506	-5.47735	-5.69035	-5.63997	-5.65395	-5.62654
**3syr**	**6**	-9.9	-9.85903	-9.873	-9.85512	-9.85572	-9.85741	-9.85799
**3ui7**	**6**	-8.25	-8.2445	-8.20235	-8.24405	-8.24221	-8.24405	-8.24092
**3uo4**	**6**	-10.51	-9.85932	-9.86061	-10.6259	-10.1595	-10.1499	-10.0668
**4bkt**	**6**	-6.3	-6.31962	-6.32087	-6.31832	-6.3183	-6.31804	-6.31904
**4ivd**	**6**	-9.2	-8.80283	-8.25284	-9.23258	-9.0287	-9.08683	-9.09033
**4jxs**	**6**	-7.76	-7.17632	-6.77967	-7.6696	-7.59889	-7.60131	-7.51106
**4rfm**	**6**	-11.28	-11.3399	-11.3128	-11.2247	-11.3107	-11.3558	-11.316
**4twp**	**6**	-10.22	-10.3481	-10.0938	-10.2065	-10.19	-10.2094	-10.1525
**5dwr**	**6**	-10.8	-10.7518	-10.8365	-10.8357	-10.8365	-10.7493	-10.8365
**Average**		**-8.89071**	**-8.74609**	**-8.65794**	**-8.88882**	**-8.83707**	**-8.83626**	**-8.83588**
**1nc1**	**7**	-11.9	-11.9089	-11.9087	-11.9061	-11.9077	-8.32361	-11.9095
**1y6r**	**7**	-8.3	-8.32621	-8.32028	-8.3265	-8.32337	-9.66642	-8.32543
**1yc1**	**7**	-9.7	-9.65148	-9.64567	-9.66855	-9.66789	-9.81279	-9.66953
**1z9g**	**7**	-9.8	-9.81272	-9.8081	-9.81302	-9.82651	-6.14816	-9.80513
**2fvd**	**7**	-6.49	-6.06435	-5.8485	-6.61097	-6.1458	-8.93241	-6.10223

**2vvn**	**7**	-9.6	-8.69969	-8.64405	-9.06458	-8.91618	-7.0978	-9.00642
**2w66**	**7**	-7.11	-6.99337	-6.86672	-7.10967	-7.10615	-7.82905	-7.10472
**2wvt**	**7**	-7.8	-7.6917	-7.83118	-7.83266	-7.83004	-6.25206	-7.82426
**2x00**	**7**	-6.3	-6.25409	-6.1475	-6.30989	-6.30046	-11.9487	-6.28582
**2xbv**	**7**	-11.94	-11.9485	-11.9518	-11.9509	-11.9483	-11.4615	-11.9492
**2xii**	**7**	-11.55	-11.1617	-9.49142	-11.4898	-10.9771	-10.0228	-11.4661
**2zb1**	**7**	-10	-8.40651	-8.87047	-10.0237	-9.78651	-10.654	-10.023
**3b65**	**7**	-10.69	-10.6446	-10.5276	-10.6534	-10.6595	-10.817	-10.6463
**3cj4**	**7**	-10.9	-10.8232	-10.8453	-10.9075	-10.8695	-7.39012	-10.8391
**3e92**	**7**	-7.4	-7.37826	-7.07853	-7.39488	-7.38146	-11.9082	-7.39117
**3ehy**	**7**	-7.1	-7.02351	-6.95772	-7.0808	-7.03771	-7.05969	-7.08407
**3fv2**	**7**	-10	-9.97594	-9.97331	-9.97726	-9.97733	-9.97922	-9.97833
**3g2n**	**7**	-9.39	-9.34365	-9.33366	-9.35281	-9.35382	-9.35264	-9.35261
**3nq9**	**7**	-4.69	-4.63875	-4.50094	-4.98555	-4.74939	-4.80566	-4.71219
**3up2**	**7**	-9.59	-9.36241	-9.31718	-9.5815	-9.57333	-9.27174	-9.62407
**4ciw**	**7**	-8.3	-8.27404	-8.27143	-8.26979	-8.27202	-8.27137	-8.27477
**4de2**	**7**	-8.01	-7.81825	-7.28965	-7.99573	-7.68162	-7.81952	-7.95532
**4djv**	**7**	-9.6	-9.58869	-9.23744	-9.59389	-9.60469	-9.58446	-9.59814
**4eor**	**7**	-9.3	-9.15954	-9.13632	-9.28007	-9.23989	-9.26765	-9.24793
**4f2w**	**7**	-9.1	-9.07128	-9.07518	-9.07611	-9.07319	-9.06873	-9.06942
**4j3l**	**7**	-11.1	-11.0828	-10.8813	-11.1287	-11.1203	-11.111	-11.1181
**4jfs**	**7**	-6.7	-6.66885	-6.69355	-6.67019	-6.67331	-6.67491	-6.67124
**4jia**	**7**	-8.96	-8.8085	-8.82962	-8.98562	-8.88692	-8.80071	-8.79059
**4lzs**	**7**	-5.6	-5.55197	-5.62659	-5.57974	-5.57877	-5.58377	-5.58312
**Average**		**-8.85931**	**-8.69425**	**-8.58309**	**-8.84896**	**-8.77479**	**-8.7902**	**-8.80717**
**1bzc**	**8**	-9.58	-9.2907	-9.29824	-9.57493	-9.54229	-9.48724	-9.57632
**1sqa**	**8**	-9.15	-9.0641	-8.64812	-9.08128	-8.96849	-9.02452	-9.08259
**1ydt**	**8**	-9.43	-8.83232	-8.83935	-9.41968	-8.99485	-8.94906	-9.07899
**1z95**	**8**	-10.51	-10.5322	-10.5595	-10.5019	-10.5082	-10.515	-10.545
**2br1**	**8**	-8.39	-8.34049	-8.21989	-8.35607	-8.32121	-8.30419	-8.32804
**2cet**	**8**	-9.3	-8.99499	-8.61572	-9.28684	-9.02257	-8.97075	-9.12994
**2qbr**	**8**	-8.43	-7.55862	-7.27058	-8.36568	-7.90792	-7.93207	-7.83601
**2v7a**	**8**	-9.79	-9.71359	-9.69033	-9.76812	-9.76112	-9.75336	-9.74856
**2w4x**	**8**	-7.3	-6.86745	-6.45385	-7.30803	-7.30817	-7.31154	-7.30589
**2xb8**	**8**	-10.8	-10.7797	-10.7737	-10.7783	-10.777	-10.7811	-10.7749
**2xdl**	**8**	-6.1	-6.08654	-6.07731	-6.08684	-6.08565	-6.08682	-6.09063
**2zcq**	**8**	-9.64	-9.4346	-9.3216	-9.63721	-9.4555	-9.45424	-9.48936
**2zy1**	**8**	-9.67	-9.38045	-9.19475	-9.55818	-9.38109	-9.42659	-9.4942
**3aru**	**8**	-6.07	-5.85683	-5.7791	-6.09739	-6.01568	-6.0345	-6.02492
**3b5r**	**8**	-11.28	-11.3151	-11.2912	-11.2662	-11.2913	-11.2999	-11.2608
**3fv1**	**8**	-9.84	-9.84893	-9.84318	-9.85249	-9.8458	-9.84147	-9.84296
**3ge7**	**8**	-10.5	-10.3866	-10.2807	-10.5298	-10.5195	-10.1626	-10.4983
**3ivg**	**8**	-10.1	-9.95919	-9.6844	-10.0997	-10.0081	-10.0418	-10.057
**3jvs**	**8**	-8	-8.02084	-7.88946	-8.01151	-8.0165	-8.00813	-8.01404
**3mss**	**8**	-7.2	-6.95259	-6.47069	-7.17694	-6.9617	-6.93524	-6.98138
**3n76**	**8**	-11.49	-11.3717	-11.3832	-11.4215	-11.3931	-11.4035	-11.3967
**3n86**	**8**	-12	-11.9754	-11.9784	-12.0098	-12.0059	-12.0104	-12.0035
**3p5o**	**8**	-8.8	-8.81417	-8.61884	-8.81687	-8.81087	-8.8114	-8.81078
**4dld**	**8**	-8.33	-8.10177	-7.99923	-8.37988	-8.41964	-8.46283	-8.40664
**4qd6**	**8**	-8.5	-7.60143	-7.32223	-8.38858	-7.93403	-8.00245	-7.91897
**4wiv**	**8**	-7.52	-7.49565	-7.40844	-7.55207	-7.54843	-7.54801	-7.53493
**Average**		**-9.14308**	**-8.94523**	**-8.80431**	**-9.12791**	**-9.03095**	**-9.02149**	**-9.04736**

**1k1i**	**9**	-9.4	-8.85392	-6.81535	-9.42281	-9.33207	-9.25385	-9.21668
**1mq6**	**9**	-9.1	-7.97378	-7.56691	-9.10487	-8.79442	-8.27328	-9.18112
**1nvq**	**9**	-11	-10.5388	-10.6745	-11.0075	-10.9777	-10.9583	-10.9957
**1o0h**	**9**	-7.38	-6.68022	-6.48533	-7.3113	-7.06881	-6.88655	-7.07213
**2y5h**	**9**	-8.07	-7.08436	-6.78019	-8.02842	-7.84423	-7.8312	-7.91711
**2zda**	**9**	-9.8	-9.71974	-9.02435	-9.79805	-9.7812	-9.78372	-9.8035
**3e5a**	**9**	-9.29	-9.2561	-8.79544	-9.30544	-9.29906	-9.33739	-9.33466
**3gnw**	**9**	-11.9	-11.5128	-10.834	-11.863	-11.8669	-11.8609	-11.8696
**3u9q**	**9**	-5.97	-5.75348	-5.65895	-6.00385	-5.87555	-5.87686	-5.84438
**3wz8**	**9**	-9.67	-9.52732	-8.84898	-9.61244	-9.5107	-9.51965	-9.60745
**4cra**	**9**	-10.01	-9.78162	-9.35427	-10.0355	-9.93149	-10.0466	-9.95451
**4crc**	**9**	-10.28	-10.098	-9.1046	-10.1858	-10.2842	-10.2022	-10.1981
**4f3c**	**9**	-9.23	-9.51842	-9.525	-9.53531	-9.56602	-9.55466	-9.54251
**4ty7**	**9**	-9.89	-8.74073	-8.65331	-9.87669	-9.72367	-9.79534	-9.91904
**4w9i**	**9**	-8.21	-8.03223	-7.28034	-8.28248	-8.23749	-8.26265	-8.26237
**4x6p**	**9**	-10.97	-9.61853	-8.98706	-10.4523	-10.7627	-10.6461	-10.944
**Average**		**-9.38563**	**-8.91813**	**-8.39929**	**-9.36412**	**-9.30351**	**-9.25558**	**-9.35393**
**1z6e**	**10**	-10.6	-9.20324	-7.75206	-10.1546	-10.5722	-10.5749	-10.5601
**2qbp**	**10**	-9.24	-7.65925	-7.30091	-9.30579	-8.8589	-8.92089	-9.04894
**2qnq**	**10**	-10.3	-10.3253	-10.0307	-10.3252	-10.3267	-10.3241	-10.3244
**3arq**	**10**	-10.68	-10.1466	-10.2676	-10.6827	-10.5164	-10.5806	-10.6021
**3ejr**	**10**	-8.48	-7.0598	-7.24718	-8.51997	-8.0984	-8.45703	-8.32564
**3nx7**	**10**	-6.96	-6.67223	-6.49396	-6.93889	-6.71907	-6.67865	-6.62326
**3oe4**	**10**	-10.6	-7.41483	-8.37713	-10.5914	-10.1405	-9.67159	-10.5822
**3ozt**	**10**	-10.7	-9.03406	-9.73413	-10.7121	-10.701	-10.6917	-10.6477
**3zdg**	**10**	-5.2	-5.15984	-5.17233	-5.16467	-5.16752	-5.16674	-5.16258
**3zsx**	**10**	-7.78	-7.37424	-7.01964	-8.04837	-7.97284	-7.98541	-7.95364
**4w9c**	**10**	-7.4	-6.9232	-6.08717	-7.22885	-7.33915	-7.33109	-7.32643
**Average**		**-8.90364**	**-7.9066**	**-7.77116**	**-8.87932**	**-8.76479**	**-8.76207**	**-8.83246**
**1vso**	**11**	-6.83	-6.49424	-6.27043	-6.82853	-6.81649	-6.8015	-6.81779
**2qbq**	**11**	-7.71	-5.81964	-6.00045	-7.665	-7.11274	-6.93276	-6.99714
**2vw5**	**11**	-8.52	-9.03236	-9.02774	-9.0388	-9.03494	-9.03168	-9.03229
**2wca**	**11**	-8.3	-7.94507	-7.72154	-8.30983	-7.97531	-8.01828	-7.99725
**2yfe**	**11**	-7.4	-7.40348	-7.36367	-7.41197	-7.40634	-7.41654	-7.42215
**2zcr**	**11**	-10.08	-10.042	-10.0137	-10.1162	-10.0687	-10.0388	-10.0696
**3b68**	**11**	-11.11	-11.1739	-11.1763	-11.1547	-11.1373	-11.1567	-11.1745
**3nw9**	**11**	-11.71	-9.68882	-8.85172	-11.7335	-11.6681	-11.1698	-11.5934
**3oe5**	**11**	-10.1	-8.36979	-7.31581	-10.1272	-9.94567	-9.83182	-9.89957
**3ozs**	**11**	-10.45	-8.69378	-9.4264	-10.4692	-10.0958	-10.4289	-10.0943
**3ueu**	**11**	-6.32	-6.11882	-6.0824	-6.36681	-6.22647	-6.26598	-6.16176
**4agn**	**11**	-6	-5.02144	-5.04407	-5.98785	-6.08385	-5.94725	-6.09683
**4eky**	**11**	-9.84	-9.84582	-9.80156	-9.85035	-9.82884	-9.84125	-9.83885
**5c2h**	**11**	-9.62	-9.06946	-8.52899	-9.63074	-9.49423	-8.92977	-9.52976
**Average**		**-8.85643**	**-8.19418**	**-8.04463**	**-8.90648**	**-8.77819**	**-8.70079**	**-8.76608**
**2p4y**	**12**	-10.86	-10.9665	-10.9187	-10.9601	-10.9616	-10.9714	-10.9749
**2wbg**	**12**	-8.4	-7.6839	-8.10459	-8.31501	-8.25317	-8.11246	-8.2302
**3b1m**	**12**	-10.12	-10.0933	-10.1135	-10.1354	-10.0494	-10.0682	-10.0471
**4agp**	**12**	-6.45	-5.29371	-5.07565	-6.30767	-6.4237	-6.20686	-6.50062
**4agq**	**12**	-6.68	-5.47185	-4.62657	-6.64214	-6.32203	-6.11708	-6.50358

**4ea2**	**12**	-8.2	-7.97538	-7.96891	-8.15826	-8.03259	-7.99677	-8.04525
**4eo8**	**12**	-6.99	-6.92441	-6.69272	-6.95176	-6.9387	-6.92393	-6.94616
**Average**		**-8.24286**	**-7.77273**	**-7.64295**	**-8.21004**	**-8.14016**	**-8.05668**	**-8.17825**
**1h22**	**13**	-11.39	-9.79514	-9.635	-10.8456	-10.2096	-10.354	-10.6153
**2fxs**	**13**	-6.7	-6.2299	-5.8013	-6.62736	-6.60545	-6.497	-6.59784
**2yge**	**13**	-8.41	-8.43032	-7.90143	-8.43361	-8.43749	-8.43458	-8.4343
**3coz**	**13**	-10	-9.89936	-9.86962	-10.0044	-10.0001	-10.0186	-10.005
**3kwa**	**13**	-4.26	-3.80517	-3.87919	-4.35523	-3.99806	-3.96974	-4.00714
**3myg**	**13**	-7.41	-7.03088	-6.63351	-7.38454	-7.30927	-7.27886	-7.3618
**3uev**	**13**	-6.6	-6.31487	-6.32101	-6.61006	-6.41329	-6.45805	-6.48362
**4cig**	**13**	-7.83	-6.99577	-6.77245	-7.51099	-7.1603	-7.28241	-7.42645
**4w9h**	**13**	-8.08	-7.73367	-7.22236	-8.10604	-8.10042	-8.05578	-8.02256
**5a7b**	**13**	-6.7	-5.81691	-5.81597	-6.71368	-6.44244	-6.30951	-6.67498
**Average**		**-7.738**	**-7.2052**	**-6.98518**	**-7.65915**	**-7.46763**	**-7.46585**	**-7.56291**
**1g2k**	**14**	-11.39	-11.5277	-11.5562	-11.4263	-11.4908	-11.6164	-11.5203
**1lpg**	**14**	-9.3	-8.21807	-8.16114	-9.11595	-9.10283	-8.67034	-8.88857
**1qf1**	**14**	-7.93	-7.71902	-7.09365	-7.95866	-7.94665	-7.92483	-7.93657
**3coy**	**14**	-10	-9.90927	-9.73016	-10.0212	-10.0275	-10.0198	-10.0217
**4gr0**	**14**	-11.69	-10.7878	-10.071	-11.6767	-11.0313	-11.0962	-11.1789
**Average**		**-10.062**	**-9.63236**	**-9.32242**	**-10.0398**	**-9.91982**	**-9.86551**	**-9.90921**
**1a30**	**15**	-7.01	-6.54055	-6.39915	-7.04512	-6.84088	-6.77916	-6.80272
**1h23**	**15**	-11.48	-9.7268	-9.59263	-10.6875	-10.2248	-10.5517	-10.4025
**3arp**	**15**	-9.64	-8.44224	-8.72367	-9.1385	-8.94367	-8.91916	-8.92076
**3tsk**	**15**	-10.34	-8.86927	-9.18783	-10.3676	-9.54804	-9.18355	-9.68072
**3uew**	**15**	-6.66	-6.26372	-6.04818	-6.69985	-6.37838	-6.40299	-6.39766
**3zso**	**15**	-8.23	-7.1262	-6.68517	-7.67393	-8.1374	-7.80252	-8.02277
**4ogj**	**15**	-7.5	-6.39964	-6.44449	-7.32713	-7.00069	-6.73759	-6.90363
**Average**		**-8.69429**	**-7.62406**	**-7.58302**	**-8.41995**	**-8.15341**	**-8.05381**	**-8.16153**
**1u1b**	**16**	-8.88	-7.72403	-7.43284	-8.81483	-8.35572	-8.00758	-8.10977
**3bv9**	**16**	-8.45	-8.20062	-8.01275	-8.4	-8.34623	-8.40646	-8.26239
**3utu**	**16**	-10.37	-7.44688	-7.20214	-9.7956	-8.70549	-8.62518	-8.87726
**Average**		**-9.23333**	**-7.79051**	**-7.54924**	**-9.00348**	**-8.46915**	**-8.3464**	**-8.41647**
**1eby**	**17**	-12.3	-12.2848	-12.24	-12.2887	-12.288	-12.2913	-12.2916
**3o9i**	**17**	-9.84	-9.82637	-9.73357	-9.85004	-9.85681	-9.85151	-9.84509
**3pww**	**17**	-10.85	-10.8149	-10.4808	-10.7887	-10.8524	-10.8248	-10.8466
**3uex**	**17**	-7.33	-6.94527	-6.81227	-7.40078	-7.05909	-6.96191	-6.97528
**4tmn**	**17**	-7.99	-7.37596	-7.27097	-8.6229	-8.38573	-8.1515	-8.6337
**Average**		**-9.662**	**-9.44946**	**-9.30753**	**-9.79022**	**-9.6884**	**-9.61621**	**-9.71845**
**5tmn**	**18**	**-8.59**	**-7.32244**	**-7.96168**	**-8.89372**	**-8.61936**	**-8.67471**	**-8.90335**
**4w9l**	**19**	**-7.9**	**-7.78076**	**-7.61016**	**-7.8647**	**-7.91336**	**-7.89053**	**-7.89637**
**2vkm**	**20**	**-11.37**	**-11.1374**	**-10.0048**	**-11.3854**	**-11.1913**	**-11.1976**	**-11.2348**
**4gid**	**23**	**-10.71**	**-11.0796**	**-11.063**	**-11.1764**	**-11.1715**	**-11.1616**	**-11.1778**
**3prs**	**24**	**-9.18**	**-8.73614**	**-8.62536**	**-9.24632**	**-9.20561**	**-9.23789**	**-9.22512**
**3uri**	**33**	**-7.85**	**-7.95376**	**-7.15295**	**-7.80549**	**-7.77117**	**-7.63163**	**-7.75399**
**3ag9**	**36**	**-7.99**	**-5.19405**	**-4.99338**	**-6.45306**	**-5.7326**	**-5.38231**	**-5.45766**

Note: The pdb codes shown in Figure 4 are 3arv, 1q8t, 3nx7 and 3uew. Their ligands have different flexibilities, i.e., 1, 5, 10 and 15 torsions, respectively, which can make docking tests more representative. The visualization of 3arv pdb code is the Chitinase A as protein complexed with the crystallization and docking results of Sanguinarine ; The visualization of 1q8t pdb code is the cAMP-dependent Protein Kinase complexed with the crystallization and docking results of Rho-kinase Inhibitor Y-27632 ; The visualization of 3nx7 pdb code is the human MMP12 as target complexed with the crystallization and docking results of an acetamide inhibitor; The visualization of 3uew pdb code is the Bovine beta-lactoglobulin as protein complexed with the crystallization and docking results of palmitic acid.

Table S2. The average RMSD of 285 test cases obtained by all compared docking programs

		**RMSD (Å)**						
**PDB**	**ntor**	**Vina**	**PSO-c4**	**PSOVina**	**GWOVina**	**MPSOVina**	**MRDPSO-c4**	**MRDPSOVina**
**3jya**	**0**	4.5286	4.53175	4.5888	4.5289	4.52926	4.52804	4.60274
**3udh**	**0**	0.21232	0.21152	0.21165	0.21206	0.21217	0.6142	0.21176
**4kzu**	**0**	0.19342	0.20145	0.21355	0.1999	0.21544	0.19727	0.20983
Average		**1.64478**	**1.64824**	**1.671333**	**1.646953**	**1.65229**	**1.779837**	**1.674777**
**1bcu**	**1**	0.46117	0.46324	0.46162	0.46266	0.4612	0.4616	0.47236
**1gpn**	**1**	4.42468	4.42469	4.42467	4.42425	4.4247	4.42469	4.42471
**2xys**	**1**	0.44453	0.44803	0.44724	0.44783	0.4455	0.44917	0.44764
**3arv**	**1**	7.77882	7.04284	7.51012	7.06648	7.64433	7.66039	6.3522
**3rsx**	**1**	0.48803	1.07702	2.71351	1.07779	0.49574	1.07581	0.48978
**4ddk**	**1**	4.41807	4.39193	4.36039	4.83473	4.83086	4.39644	4.83018
Average		**3.00255**	**2.974625**	**3.319592**	**3.05229**	**3.050388**	**3.078017**	**2.836145**
**1c5z**	**2**	0.50284	0.37733	0.37781	1.13583	0.37799	0.50298	0.37607
**1o5b**	**2**	1.02205	0.91278	0.91278	1.02136	1.02191	1.02201	1.03729
**1r5y**	**2**	0.22276	1.65597	2.25378	0.22212	0.22247	0.22038	0.71413
**1s38**	**2**	0.13502	0.13807	1.29537	0.13908	0.13802	0.71392	0.13563
**2iwx**	**2**	0.5695	0.56945	0.5695	0.56938	0.56945	0.5694	0.56935
**2weg**	**2**	1.34197	1.45329	2.4006	1.34797	1.51445	1.902	1.51867
**2yki**	**2**	2.19573	0.23015	0.23846	0.25544	0.2423	0.23449	0.23954
**3ary**	**2**	5.2844	5.99158	5.69327	5.31545	5.54624	5.73446	6.19112
**3g2z**	**2**	4.96368	4.49492	4.52499	4.72888	4.8269	4.68976	4.47988
**3gv9**	**2**	4.37868	1.8957	3.65574	4.37988	4.00023	3.57804	4.11055
**3kr8**	**2**	0.90477	0.56432	0.55753	0.90524	0.68027	0.78753	0.73082
**3pxf**	**2**	9.46707	9.37744	9.28807	9.33637	9.34681	9.31641	9.42736
**3twp**	**2**	9.34878	6.53269	9.9334	9.33972	8.7688	8.84763	8.22884
**3u5j**	**2**	0.22816	0.2282	0.22822	0.22787	0.22817	0.81259	0.22829
**3zt2**	**2**	0.7128	0.48273	0.77787	0.71316	0.71283	0.67403	0.67331
**4f09**	**2**	0.24435	0.22987	0.23323	0.23613	0.23526	0.23174	0.22969
**4gfm**	**2**	6.4969	6.4643	6.49071	6.49886	6.48109	6.46374	6.521
**4jsz**	**2**	11.30485	11.28977	10.40552	11.31385	11.12875	11.18049	10.45838
**4k77**	**2**	3.0052	2.91859	3.55542	2.66295	2.94281	2.94238	2.82892
**4kzq**	**2**	0.22941	0.22959	0.41594	0.22928	0.23211	0.24092	0.23198
**4mme**	**2**	0.40994	0.42001	0.41552	0.41195	0.41404	0.4165	0.41555
**4owm**	**2**	2.87813	2.71212	4.85434	2.87065	2.86522	2.78415	2.83777
**4u4s**	**2**	9.23703	8.18462	8.18304	9.23386	9.2323	8.6831	8.37942
Average		**3.264523**	**2.928413**	**3.359179**	**3.178056**	**3.118627**	**3.154289**	**3.067981**

**1e66**	3	0.3854	0.38638	0.38435	0.3896	0.38648	0.38791	0.38776
**1gpk**	3	0.22189	0.22189	0.22189	0.22197	0.22189	0.22186	0.22186
**1qkt**	3	0.90469	0.90481	0.90487	0.90498	0.90532	0.90666	0.90474
**1uto**	3	2.20172	1.06968	2.39818	1.02769	1.09121	1.04461	1.01903
**1ydr**	3	0.53186	0.52751	0.85777	0.52873	0.53054	0.53369	0.52901
**2pog**	3	0.37153	0.37188	0.37217	0.37165	0.37121	0.37162	0.37183
**2wer**	3	0.5368	0.5368	0.5368	0.53665	0.5368	0.5368	0.5368
**3acw**	3	1.39846	1.3606	1.43319	1.36117	1.32418	1.32374	1.32552
**3ao4**	3	1.61486	1.78815	2.76403	1.61282	1.48934	1.49369	1.84524
**3bgz**	3	1.05709	0.93617	0.88783	1.058	0.9972	1.05783	0.99796
**3g31**	3	3.59422	3.75698	3.6634	3.5941	3.59902	3.89666	3.82395
**3gy4**	3	1.58908	1.10394	0.98374	1.90434	1.21493	1.252	1.20792
**3kgp**	3	1.97869	1.35008	1.3268	1.87578	1.5358	1.52149	1.46911
**3pyy**	3	0.92037	0.54312	1.08672	0.99559	1.22624	0.47049	0.39756
**3wtj**	3	1.31817	0.81101	0.82202	1.31849	1.21638	1.67852	1.11129
**4cr9**	3	4.77142	4.77856	4.3702	4.77217	4.77767	4.35408	4.7698
**4de1**	3	1.19874	2.72623	6.54208	1.10395	2.77597	1.89046	2.79007
**4de3**	3	2.78122	0.46676	1.58274	5.94763	0.50057	0.63794	1.68422
**4e6q**	3	1.10289	0.93307	0.90567	1.11763	1.12518	1.11227	1.10831
**4ih5**	3	2.01441	2.88695	3.50305	1.89346	3.27269	2.70341	2.42568
**4j21**	3	0.20251	0.2025	0.2025	0.2025	0.20268	0.20355	0.20249
**4llx**	3	6.28084	10.63988	9.08407	10.7246	9.95499	9.69309	8.47368
**4m0y**	3	6.98409	6.88052	6.72732	6.98582	6.98334	6.93998	7.0111
**5c28**	3	2.51949	2.98614	2.15982	2.52033	2.51795	2.55135	2.51972
**Average**		**1.936685**	**2.007067**	**2.238384**	**2.207069**	**2.031566**	**1.949321**	**1.963944**
**1q8u**	4	0.2952	0.29545	0.29566	0.29619	0.29566	0.29586	0.29619
**1syi**	4	0.78277	0.59247	0.65646	0.78277	0.78282	0.71889	0.78303
**2al5**	4	0.2225	0.22644	0.28717	0.2275	0.22592	0.22672	0.22513
**2cbv**	4	1.18177	1.46641	1.18159	0.33454	0.33432	1.18187	1.18257
**2hb1**	4	4.22938	1.78403	2.6057	3.8517	1.77653	3.1123	2.46276
**2j7h**	4	2.17399	2.17204	2.54141	2.16903	2.16978	2.16736	2.17469
**2v00**	4	1.34352	1.01632	1.94185	1.44886	1.22981	1.17933	1.36803
**2wnc**	4	2.85702	2.85373	3.05361	2.91216	2.89911	2.87587	2.86908
**2wtv**	4	1.79661	1.63603	1.61779	1.72602	1.71546	1.79675	1.72591
**2ymd**	4	1.09888	0.23963	0.23917	0.23103	0.23354	0.23344	0.23372
**3b27**	4	0.40877	0.40895	1.26776	0.40458	0.41018	0.40749	0.40797
**3dx1**	4	0.45284	1.18191	0.87272	0.45216	0.45214	0.66234	0.45229
**3f3a**	4	1.67715	1.56843	1.48704	1.67705	1.68571	1.59996	1.58102
**3f3c**	4	0.89442	0.36586	0.36461	0.89422	0.56371	0.69613	0.63014
**3g0w**	4	0.17916	0.17909	0.18109	0.17944	0.17939	0.17878	0.1803
**3gr2**	4	9.00632	10.25825	8.94994	8.76126	8.39146	9.43999	9.80492
**3lka**	4	5.23287	5.3476	5.31303	5.25343	5.18374	5.17975	5.3263
**3n7a**	4	0.3029	0.24456	0.23893	0.2533	0.21784	0.24939	0.24334
**3qqs**	4	5.02439	2.13272	2.36324	5.03672	3.40121	2.40344	2.54528
**3rr4**	4	0.4496	0.91093	2.93114	0.427	0.47174	0.46957	0.50894
**3u8k**	4	0.77728	0.75693	0.75714	0.77851	0.82604	0.8378	0.83383
**3u8n**	4	0.37261	0.36339	0.37236	0.37299	0.37337	0.3761	0.37836
**4abg**	4	1.41255	0.94121	0.84195	1.41358	1.77433	0.99222	1.02836
**4ddh**	4	1.55767	1.45579	1.30448	1.55561	1.50234	1.56081	1.56138
**4dli**	4	0.52989	0.52656	0.52677	0.52896	0.52513	0.52434	0.52747
**4hge**	4	1.30311	0.9958	1.65828	1.20805	1.30185	1.38848	0.9164

**4ivb**	**4**	0.23434	0.23624	0.23638	0.23466	0.23505	0.23731	0.23585
**4j28**	**4**	1.00239	1.00061	1.00868	1.00382	1.00259	1.20022	1.00049
**Average**		**1.671425**	**1.469906**	**1.61057**	**1.586255**	**1.434313**	**1.506875**	**1.481563**
**1nc3**	**5**	0.18233	0.22667	0.25143	0.18221	0.18388	0.24889	0.18432
**1o3f**	**5**	1.55459	1.91045	6.25815	1.533	1.53654	2.5194	1.55063
**1oyt**	**5**	0.26459	0.26524	0.55058	0.2636	0.26302	0.26489	0.2625
**1p1n**	**5**	1.22226	0.88996	0.86073	1.22273	0.98274	0.86126	0.86392
**1p1q**	**5**	4.85901	3.63811	4.57653	4.85912	4.86622	5.10061	4.60331
**1ps3**	**5**	0.48549	0.70009	0.48452	0.48337	0.4824	0.48482	0.48363
**1q8t**	**5**	7.69295	7.74457	7.71577	7.67984	7.74494	7.73735	7.18478
**2r9w**	**5**	7.38732	6.57263	6.38475	7.40428	7.28941	7.2686	6.65055
**2wn9**	**5**	7.21338	6.73333	6.51904	7.21204	6.79956	7.03743	7.09944
**2xj7**	**5**	0.28853	0.28547	0.28899	0.28834	0.28817	0.28827	0.28626
**3d4z**	**5**	0.94002	3.53802	2.8398	0.95145	0.93258	1.80944	0.94716
**3d6q**	**5**	7.02634	3.06929	5.18596	5.23573	3.88397	4.44927	2.93393
**3dd0**	**5**	1.70595	1.83724	3.37157	1.17943	2.22897	1.70469	2.22759
**3fcq**	**5**	9.39327	5.56845	3.5869	3.0742	3.05787	2.7894	3.78015
**3fur**	**5**	1.60751	0.99882	1.11822	0.88236	0.87369	0.87914	0.88293
**3gbb**	**5**	0.77196	0.58307	0.65556	0.80216	0.68571	0.74955	0.79186
**3jvr**	**5**	0.69396	3.06163	4.54165	0.69219	0.59886	0.72557	0.62422
**3qgy**	**5**	6.19046	1.93551	2.81212	6.19784	6.23152	5.72075	5.68124
**3rlr**	**5**	0.28464	0.9516	0.62338	0.28181	0.29868	0.617	0.2924
**3ryj**	**5**	1.47718	1.207	1.7363	1.42362	1.27954	0.95811	1.03351
**3uuo**	**5**	1.11019	1.03699	1.12141	1.10942	1.11222	1.10942	1.1115
**4e5w**	**5**	0.1477	0.45602	0.52016	0.14739	0.14721	0.14714	0.14735
**4f9w**	**5**	0.31706	0.72069	1.01147	0.27199	0.26991	0.64429	0.36275
**4gkm**	**5**	5.83666	4.41988	5.36238	5.81601	5.61666	5.25892	4.71939
**4ih7**	**5**	1.13915	0.90204	0.57743	1.05998	1.13955	0.98076	1.13935
**4ivc**	**5**	0.184	0.18176	0.28626	0.18518	0.18255	0.18381	0.18157
**4k18**	**5**	0.60979	2.25621	2.2952	0.60984	0.61294	0.70239	1.00807
**4kz6**	**5**	5.26356	1.79895	2.40705	5.2587	3.15675	3.55614	4.40819
**4m0z**	**5**	0.46942	0.45513	0.45865	0.46806	0.46725	0.46104	0.45849
**4mgd**	**5**	0.51393	0.43666	0.48328	0.87352	0.43065	0.51441	0.44303
**4pcs**	**5**	0.26784	0.2659	0.34725	0.59773	0.26804	0.35413	0.34763
**4qac**	**5**	0.47292	0.47459	0.47069	0.47459	0.47565	0.47385	0.4751
**5aba**	**5**	6.15278	6.42645	5.66897	4.35284	4.6023	3.39291	3.40584
**Average**		**2.537174**	**2.168134**	**2.465823**	**2.214381**	**2.090605**	**2.12102**	**2.017351**
**1owh**	**6**	0.3249	0.32783	0.3293	0.32705	0.32789	2.17488	0.32696
**1pxn**	**6**	2.48552	2.82506	2.1801	2.48709	2.44055	0.83728	2.29153
**1w4o**	**6**	0.84045	0.75664	0.65299	0.84131	0.80899	1.38599	0.8386
**2brb**	**6**	1.48009	1.31747	1.11967	1.4821	1.40112	1.15419	1.38526
**2c3i**	**6**	4.28865	1.14818	1.26582	5.10108	1.70462	5.28037	1.27427
**2j78**	**6**	4.90807	4.23577	5.25742	4.90865	5.24531	5.84161	5.31339
**2p15**	**6**	9.48983	3.80304	4.6053	8.57975	4.4183	0.37085	4.9278
**2qe4**	**6**	0.37528	0.36064	0.37001	0.37163	0.3717	0.57722	0.3668
**2xnb**	**6**	0.57915	0.57779	0.57781	0.57927	0.58048	1.37097	0.578
**3dx2**	**6**	1.2814	1.02089	1.50606	1.3706	1.41343	0.32479	1.36785
**3dxg**	**6**	0.32548	1.13423	2.06946	0.32441	0.59627	5.34031	0.46846
**3e93**	**6**	5.34743	4.98488	4.81875	5.34603	5.27698	0.32541	5.37676
**3ebp**	**6**	0.56778	1.06863	0.90363	0.56225	0.57712	0.57419	0.58326
**3f3d**	**6**	1.08224	1.00662	1.12021	1.04269	1.16003	1.08165	1.04113

**3f3e**	**6**	1.1415	0.94505	0.91161	1.39254	1.15043	1.29182	1.19251
**3gc5**	**6**	0.38294	0.76319	0.61311	0.38293	0.40453	0.56541	0.38231
**3k5v**	**6**	3.3836	3.27292	3.3041	3.40263	3.52935	3.43132	3.37514
**3l7b**	**6**	0.73854	0.73909	0.71408	0.73818	0.73914	0.73851	0.73868
**3r88**	**6**	12.77686	10.29806	9.49835	11.99331	11.0295	11.4074	11.16473
**3syr**	**6**	0.53472	0.53472	0.50538	0.53464	0.5348	0.53462	0.53473
**3ui7**	**6**	0.4941	0.49175	0.82199	0.48942	0.48899	0.49184	0.4909
**3uo4**	**6**	7.59781	3.8468	4.92585	8.71276	3.39828	5.43854	5.42998
**4bkt**	**6**	0.58224	0.57085	0.57402	0.58818	0.56425	0.57413	0.57731
**4ivd**	**6**	0.7292	1.57616	4.00863	0.72725	1.208	0.55829	0.62852
**4jxs**	**6**	2.6241	3.26719	4.11975	2.99891	3.24218	2.59335	1.73869
**4rfm**	**6**	4.39121	1.53261	1.88207	4.38932	2.57712	1.47343	2.25689
**4twp**	**6**	2.45562	1.10092	1.38276	2.61825	1.61578	2.01756	1.98833
**5dwr**	**6**	0.42145	0.93723	0.42141	0.42281	0.42143	0.63593	0.42143
**Average**		**2.55822**	**1.944436**	**2.159273**	**2.596966**	**2.043806**	**2.085424**	**2.037865**
**1nc1**	**7**	0.24066	0.24346	0.24363	0.24829	0.24203	0.41285	0.24747
**1y6r**	**7**	0.44136	0.41607	0.4797	0.41476	0.41586	0.55946	0.41631
**1yc1**	**7**	0.56123	0.38379	0.38464	0.55903	0.55966	0.67424	0.55931
**1z9g**	**7**	0.69094	0.63711	0.89852	0.86677	0.69115	3.09766	0.65379
**2fvd**	**7**	5.77053	2.9139	4.71954	5.12309	2.86892	3.58928	3.6917
**2vvn**	**7**	9.57708	1.05156	2.19819	4.52459	3.9147	0.26535	5.40565
**2w66**	**7**	0.25801	1.97606	3.87185	0.25954	0.25889	0.36234	0.27572
**2wvt**	**7**	0.35851	0.83096	0.35915	0.35834	0.36112	0.81171	0.3558
**2x00**	**7**	0.65732	0.7375	1.18316	0.6579	0.67021	0.58136	0.69246
**2xbv**	**7**	0.5781	0.58661	0.57783	0.57322	0.58683	0.67212	0.57752
**2xii**	**7**	0.70027	0.80084	3.89754	0.61683	1.45218	0.55866	0.62423
**2zb1**	**7**	0.55438	3.73496	3.43008	0.55852	0.98722	0.34747	0.55842
**3b65**	**7**	0.35194	0.40133	0.66284	0.34472	0.34479	0.78482	0.36458
**3cj4**	**7**	0.9757	0.85422	0.44698	0.97591	0.67306	2.29943	0.84111
**3e92**	**7**	2.31318	2.33906	3.01739	2.31282	2.3124	0.244	2.29754
**3ehy**	**7**	3.88354	1.33045	2.70446	2.85535	1.4462	0.88767	1.46771
**3fv2**	**7**	0.16258	0.16188	0.33027	0.16235	0.16245	0.1622	0.16257
**3g2n**	**7**	1.31933	1.31209	1.3906	1.31971	1.31813	1.31896	1.32209
**3nq9**	**7**	5.4585	6.6981	6.42759	7.31686	7.16904	6.80885	5.98151
**3up2**	**7**	2.66309	2.32921	3.00595	2.65292	2.44557	3.53549	2.59191
**4ciw**	**7**	0.34879	0.34409	0.39973	0.34886	0.34896	0.34391	0.34485
**4de2**	**7**	0.54618	1.42138	2.60415	0.56376	2.44103	0.70193	0.55542
**4djv**	**7**	0.39782	0.4964	1.70845	0.39443	0.38552	0.40948	0.39661
**4eor**	**7**	1.44291	2.30712	2.8152	2.63872	3.67238	3.38206	3.3404
**4f2w**	**7**	0.26163	0.25749	0.25783	0.2591	0.26222	0.25754	0.26182
**4j3l**	**7**	1.3117	1.29021	1.26819	1.3275	1.31768	1.31734	1.32388
**4jfs**	**7**	1.44812	1.37091	1.5096	1.44528	1.43245	1.44433	1.44922
**4jia**	**7**	8.77155	1.71753	2.30615	8.69786	6.34986	7.15519	5.94726
**4lzs**	**7**	0.57778	0.97093	1.17041	0.57989	0.58351	0.57857	0.57917
**Average**		**1.814577**	**1.376387**	**1.871366**	**1.68817**	**1.574966**	**1.502216**	**1.492622**
**1bzc**	**8**	1.18168	1.27754	1.77513	1.08486	0.97916	1.04735	0.97047
**1sqa**	**8**	1.95677	1.9061	2.62343	1.67183	1.89315	1.8374	1.75725
**1ydt**	**8**	4.71815	5.01687	7.19313	4.57889	5.63391	6.44029	6.08275
**1z95**	**8**	0.98762	0.70898	0.70965	0.84906	0.61606	0.75692	0.84442
**2br1**	**8**	1.93081	1.94698	1.5966	1.93226	1.94846	1.81932	1.95142
**2cet**	**8**	7.93321	3.88406	6.11139	7.93673	7.82041	7.26297	7.93634

**2qbr**	**8**	6.29572	4.94642	5.63194	6.43807	4.0395	2.4372	2.08543
**2v7a**	**8**	1.34039	0.77958	0.92687	1.16813	1.00053	0.83165	0.73698
**2w4x**	**8**	0.71503	1.24564	2.64426	0.71454	0.71478	0.71367	0.71516
**2xb8**	**8**	0.36444	0.36413	0.32703	0.36836	0.36498	0.36865	0.36591
**2xdl**	**8**	0.31951	0.31658	0.51142	0.31754	0.31695	0.31718	0.32131
**2zcq**	**8**	5.65792	5.71891	5.57502	5.6306	5.75809	5.73864	5.68189
**2zy1**	**8**	5.15022	2.38598	2.88068	2.899	2.94654	2.50345	3.14944
**3aru**	**8**	3.31789	1.79968	3.51376	3.30831	1.97934	2.33911	2.27719
**3b5r**	**8**	0.66888	0.6544	0.72762	0.67099	0.70947	0.66271	0.6913
**3fv1**	**8**	0.70276	0.44466	0.36696	0.49638	0.44521	0.34309	0.34329
**3ge7**	**8**	0.92161	1.10715	0.8748	0.92178	0.91832	1.06042	0.88523
**3ivg**	**8**	1.68838	1.52451	2.60149	1.679	1.64178	1.62529	1.49646
**3jvs**	**8**	3.01946	2.34805	3.23497	2.77201	2.50014	2.34957	2.43147
**3mss**	**8**	2.79076	2.59432	4.50898	2.79513	3.26226	2.66983	2.85134
**3n76**	**8**	1.46596	0.97354	1.12795	1.28705	1.11721	1.11725	1.13169
**3n86**	**8**	0.95558	0.90634	0.89671	0.95914	0.95811	0.95321	0.96233
**3p5o**	**8**	1.16947	1.17084	1.29415	1.17574	1.16765	1.17017	1.17175
**4dld**	**8**	1.8973	2.04796	2.9603	1.12811	0.66815	0.54735	0.59874
**4qd6**	**8**	5.07934	4.65817	5.37919	5.1733	2.59941	2.41781	2.79801
**4wiv**	**8**	1.21453	1.93269	4.38154	1.24426	1.15794	1.13468	1.2039
**Average**		**2.44013**	**2.025388**	**2.70673**	**2.276964**	**2.04452**	**1.940968**	**1.978518**
**1k1i**	**9**	4.75855	3.40564	6.35836	4.73076	4.48193	4.48188	3.65537
**1mq6**	**9**	1.3627	4.49616	4.67114	1.35284	1.95052	4.20733	1.03745
**1nvq**	**9**	5.34053	3.01967	3.78508	5.34206	5.33895	4.95344	5.34092
**1o0h**	**9**	1.39461	3.96663	5.82607	2.26493	2.94649	1.83814	1.23234
**2y5h**	**9**	2.21677	4.27262	5.28479	2.10607	1.35176	1.91968	1.96663
**2zda**	**9**	0.89595	0.49449	2.23291	0.53363	0.47085	0.49139	0.59684
**3e5a**	**9**	1.18398	1.20739	3.24625	1.14686	1.12904	1.05009	1.05769
**3gnw**	**9**	0.29316	1.06046	2.29311	0.29469	0.29582	0.29372	0.29317
**3u9q**	**9**	1.14655	0.88457	1.13842	1.13383	1.36235	1.11418	1.0313
**3wz8**	**9**	2.11855	1.8226	4.92274	2.00509	1.87043	1.82968	1.95058
**4cra**	**9**	2.15297	1.40533	4.50436	1.29636	0.43194	0.33065	0.83838
**4crc**	**9**	0.67806	1.35073	5.03315	2.56121	0.52286	1.40983	0.73989
**4f3c**	**9**	1.34634	0.4678	0.39678	0.59447	0.39836	0.37277	0.46752
**4ty7**	**9**	9.80336	6.67716	5.69553	9.79886	2.64055	3.67502	3.07386
**4w9i**	**9**	1.45983	1.31287	2.71138	1.43209	1.44113	1.35618	1.35955
**4x6p**	**9**	0.88988	3.643	6.67568	2.21373	1.73309	1.79413	0.96288
**Average**		**2.315112**	**2.467945**	**4.048484**	**2.425468**	**1.772879**	**1.944882**	**1.600273**
**1z6e**	**10**	1.18685	3.13794	7.25234	1.11378	0.36085	0.34293	0.37161
**2qbp**	**10**	1.03503	3.89592	5.00297	0.93612	1.05946	1.11179	0.81616
**2qnq**	**10**	0.48288	0.48411	2.34965	0.48399	0.48409	0.48495	0.48531
**3arq**	**10**	10.19791	3.40043	3.90753	10.19364	9.13494	9.33094	8.37431
**3ejr**	**10**	1.78485	5.64191	5.03424	1.33114	3.43925	1.55881	2.42855
**3nx7**	**10**	1.47026	1.24115	2.73731	1.34656	1.37326	1.98409	1.37474
**3oe4**	**10**	0.63582	6.34745	3.93124	0.63766	1.66254	4.32871	0.53952
**3ozt**	**10**	1.05731	4.06038	2.38057	1.04107	0.58601	0.52271	0.47046
**3zdg**	**10**	0.51626	0.51634	0.72256	0.5132	0.51399	0.51497	0.51984
**3zsx**	**10**	5.4442	3.82755	4.71521	4.31782	3.96547	3.55943	1.84238
**4w9c**	**10**	0.97269	1.11428	3.87351	1.65637	0.39846	0.61064	0.50423
**Average**		**2.253096**	**3.060678**	**3.809739**	**2.14285**	**2.088938**	**2.213634**	**1.611555**

**1vso**	**11**	1.50242	2.56596	3.12116	1.38238	1.39305	1.46593	1.53672
**2qbq**	**11**	2.17251	8.38656	8.35867	3.60895	3.3898	2.46415	2.80466
**2vw5**	**11**	1.65155	0.2072	0.21144	0.22266	0.21361	0.20934	0.20729
**2wca**	**11**	3.91884	3.84726	4.06423	3.92724	3.9666	3.78817	3.70149
**2yfe**	**11**	1.47749	1.20453	1.39538	1.48307	1.47193	1.44147	1.44564
**2zcr**	**11**	3.20755	1.71029	1.45441	2.73713	2.50355	2.17485	2.72631
**3b68**	**11**	1.07552	0.79567	1.01253	1.08871	0.80536	0.77868	0.77564
**3nw9**	**11**	0.34428	4.14505	5.89853	0.34807	0.44146	1.34721	0.55198
**3oe5**	**11**	0.68607	4.02839	6.02372	0.58666	0.66271	0.77913	0.77156
**3ozs**	**11**	0.69827	3.33288	2.06077	0.52031	1.63051	0.54104	1.61739
**3ueu**	**11**	4.35596	4.2991	3.82336	4.38155	4.46242	4.31145	5.30922
**4agn**	**11**	4.58765	4.86874	4.58869	4.62351	2.56636	2.8968	1.91326
**4eky**	**11**	0.65612	0.65611	0.6331	0.65533	0.6575	0.65575	0.65526
**5c2h**	**11**	1.52201	2.01719	4.76873	1.45511	1.10996	3.29897	1.46447
**Average**		**1.989731**	**3.004638**	**3.386766**	**1.930049**	**1.805344**	**1.868067**	**1.820064**
**2p4y**	**12**	1.38927	0.57693	0.5962	0.71767	0.68373	0.65932	0.56945
**2wbg**	**12**	2.74531	3.74911	2.61654	2.70283	3.10654	3.24061	3.15412
**3b1m**	**12**	0.70251	1.02026	0.71069	1.18929	1.35186	1.51678	1.51798
**4agp**	**12**	3.72278	6.04213	7.49918	4.33918	2.54862	2.2602	2.52818
**4agq**	**12**	2.81875	4.90779	7.25982	3.30261	2.68283	4.11794	3.46834
**4ea2**	**12**	2.4135	2.75877	2.39154	2.34184	2.25033	2.28684	2.26917
**4eo8**	**12**	0.78287	0.80168	1.50751	0.74124	0.91554	0.95914	0.74366
**Average**		**2.082141**	**2.836667**	**3.225926**	**2.190666**	**1.934207**	**2.14869**	**2.035843**
**1h22**	**13**	9.64256	3.31598	4.67747	7.32938	6.93513	6.44473	7.26746
**2fxs**	**13**	6.01932	4.91184	4.91078	5.89046	5.60782	6.07173	5.99627
**2yge**	**13**	0.5578	0.55486	1.93813	0.55618	0.55907	0.56117	0.55801
**3coz**	**13**	0.65931	1.23855	1.27833	0.67858	0.6144	0.62591	0.60005
**3kwa**	**13**	3.79003	3.93038	4.67608	3.65918	3.9876	4.18097	4.10297
**3myg**	**13**	3.02405	1.92773	6.02241	1.41102	2.2426	1.54247	0.95833
**3uev**	**13**	1.75649	2.10079	2.77857	1.76574	1.83453	2.67864	2.75985
**4cig**	**13**	6.34777	6.10362	6.11168	6.2843	5.2896	5.79614	2.46914
**4w9h**	**13**	1.5825	1.79827	3.09346	1.54667	1.56581	1.47617	1.44984
**5a7b**	**13**	3.0614	3.7829	3.85084	3.54125	2.8477	3.05287	2.30456
**Average**		**3.644123**	**2.966492**	**3.933775**	**3.266276**	**3.148426**	**3.24308**	**2.846648**
**1g2k**	**14**	0.99409	0.90261	0.89198	1.11665	1.00176	0.97449	0.95391
**1lpg**	**14**	9.54336	4.50576	4.79628	5.80945	1.12713	3.26108	3.19973
**1qf1**	**14**	0.78445	1.3336	3.43038	0.71747	0.65151	0.67852	0.76824
**3coy**	**14**	0.32919	1.09458	1.57415	0.4067	0.29055	0.28806	0.33177
**4gr0**	**14**	2.36354	2.02892	3.54389	2.21469	1.22383	1.2816	1.13808
**Average**		**2.802926**	**1.973094**	**2.847336**	**2.052992**	**0.858956**	**1.29675**	**1.278346**
**1a30**	**15**	7.37267	5.86502	5.21152	8.29383	6.18523	5.81915	6.88113
**1h23**	**15**	9.76612	6.25236	6.4895	7.4932	5.55406	6.79313	4.60313
**3arp**	**15**	10.12363	4.81637	6.90944	9.48277	10.01933	8.40873	7.87461
**3tsk**	**15**	3.83177	2.46799	3.21978	3.84614	2.27556	2.917	2.4618
**3uew**	**15**	3.85362	3.25654	4.07584	3.31679	4.26717	5.48226	2.8406
**3zso**	**15**	0.94985	4.6343	5.42365	5.91373	1.1606	3.73352	1.69536
**4ogj**	**15**	6.95353	3.32914	5.08547	7.42102	7.17069	6.04413	6.99399
**Average**		**6.121599**	**4.374531**	**5.202171**	**6.538211**	**5.233234**	**5.599703**	**4.764374**

**1u1b**	**16**	2.45255	4.07582	6.25476	4.47402	1.89108	4.85934	3.17784
**3bv9**	**16**	0.88078	1.68325	2.37323	0.88355	0.77469	0.83775	0.77129
**3utu**	**16**	5.36746	5.83622	5.98811	3.29256	3.20212	3.43651	3.15768
**Average**		**2.900263**	**3.865097**	**4.872033**	**2.883377**	**1.955963**	**3.044533**	**2.368937**
**1eby**	**17**	0.36822	0.36807	1.41598	0.36648	0.36751	0.36556	0.3694
**3o9i**	**17**	0.41138	0.41586	1.25927	0.35341	0.36372	0.36372	0.35507
**3pww**	**17**	1.97791	1.06775	3.00877	1.37583	0.8008	0.91613	0.94559
**3uex**	**17**	4.22664	3.80814	3.90297	4.36656	4.17119	4.1552	3.79085
**4tmn**	**17**	6.83212	7.14642	8.16875	3.86872	3.82819	4.97444	2.61689
**Average**		**2.763254**	**2.561248**	**3.551148**	**2.0662**	**1.906282**	**2.15501**	**1.61556**
**5tmn**	**18**	**5.40224**	**5.52766**	**5.35895**	**2.76089**	**2.48901**	**2.74153**	**2.70734**
**4w9l**	**19**	**1.3026**	**1.01213**	**1.1766**	**1.27555**	**1.30811**	**1.24374**	**1.2822**
**2vkm**	**20**	**2.33779**	**1.49402**	**2.92473**	**2.03441**	**1.78716**	**1.80564**	**1.78867**
**4gid**	**23**	**2.69205**	**0.4652**	**0.58878**	**0.73502**	**0.80647**	**0.84427**	**0.87129**
**3prs**	**24**	**2.0391**	**3.82227**	**4.43011**	**1.9732**	**1.93766**	**2.07144**	**2.02506**
**3uri**	**33**	**11.99391**	**2.04019**	**4.2325**	**9.59937**	**2.48888**	**3.60815**	**2.17813**
**3ag9**	**36**	**10.03285**	**8.67705**	**9.01488**	**8.67273**	**10.11324**	**9.07064**	**9.01759**

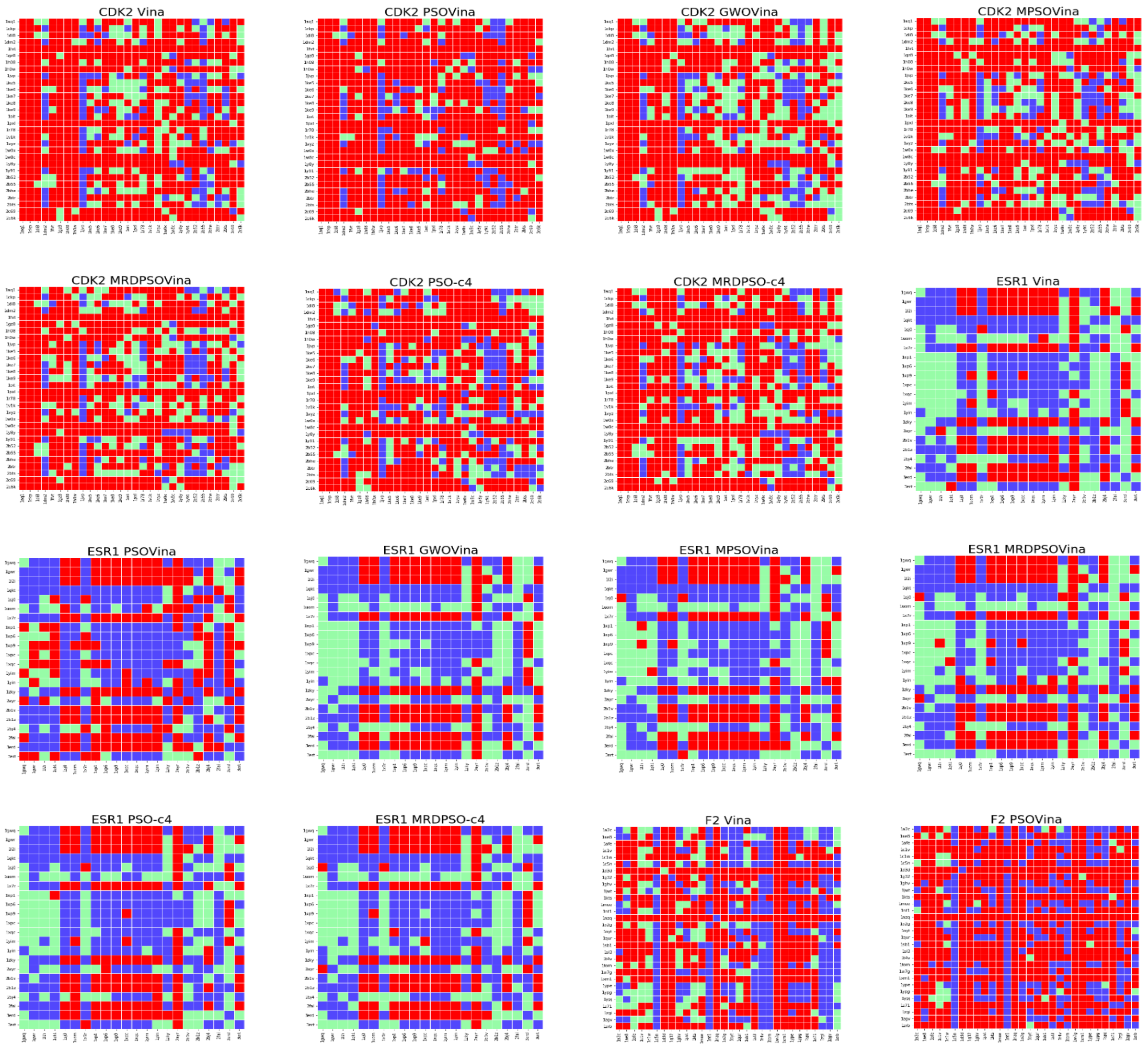

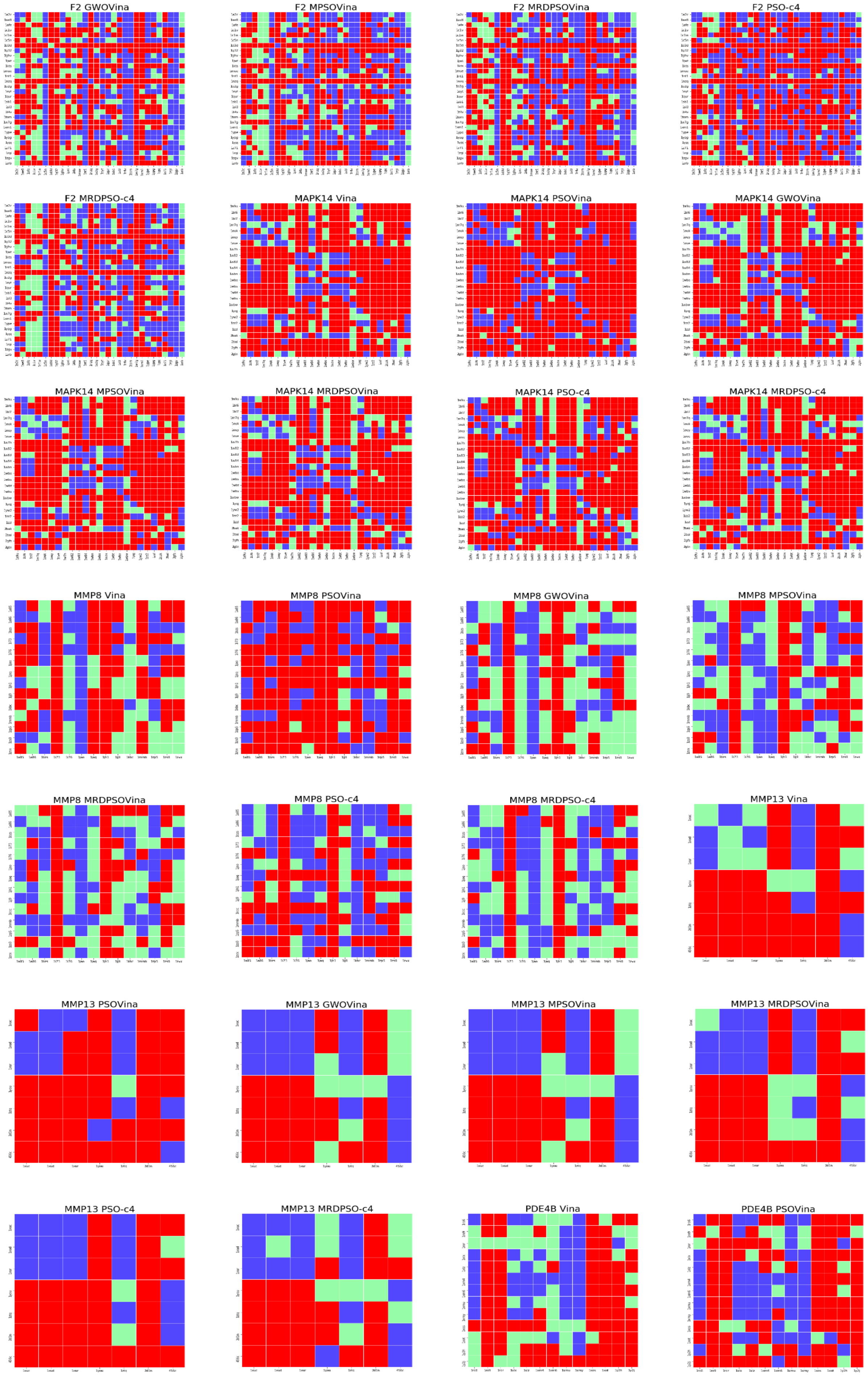
F2 GWOVina
F2 MPSOVina
F2 MRDPSOVina
F2 PSO-c4
F2 MRDPSO-c4
MAPK14 Vina
MAPK14 PSOVina
MAPK14 GWOVina
MAPK14 MPSOVina
MAPK14 MRDPSOVina
MAPK14 PSO-c4
MAPK14 MRDPSO-c4
MMP8 Vina
MMP8 PSOVina
MMP8 GWOVina
MMP8 MPSOVina
MMP8 MRDPSOVina
MMP8 PSO-c4
MMP8 MRDPSO-c4
MMP13 Vina
MMP13 PSOVina
MMP13 GWOVina
MMP13 MPSOVina
MMP13 MRDPSOVina
MMP13 PSO-c4
MMP13 MRDPSO-c4
PDE4B Vina
PDE4B PSOVina

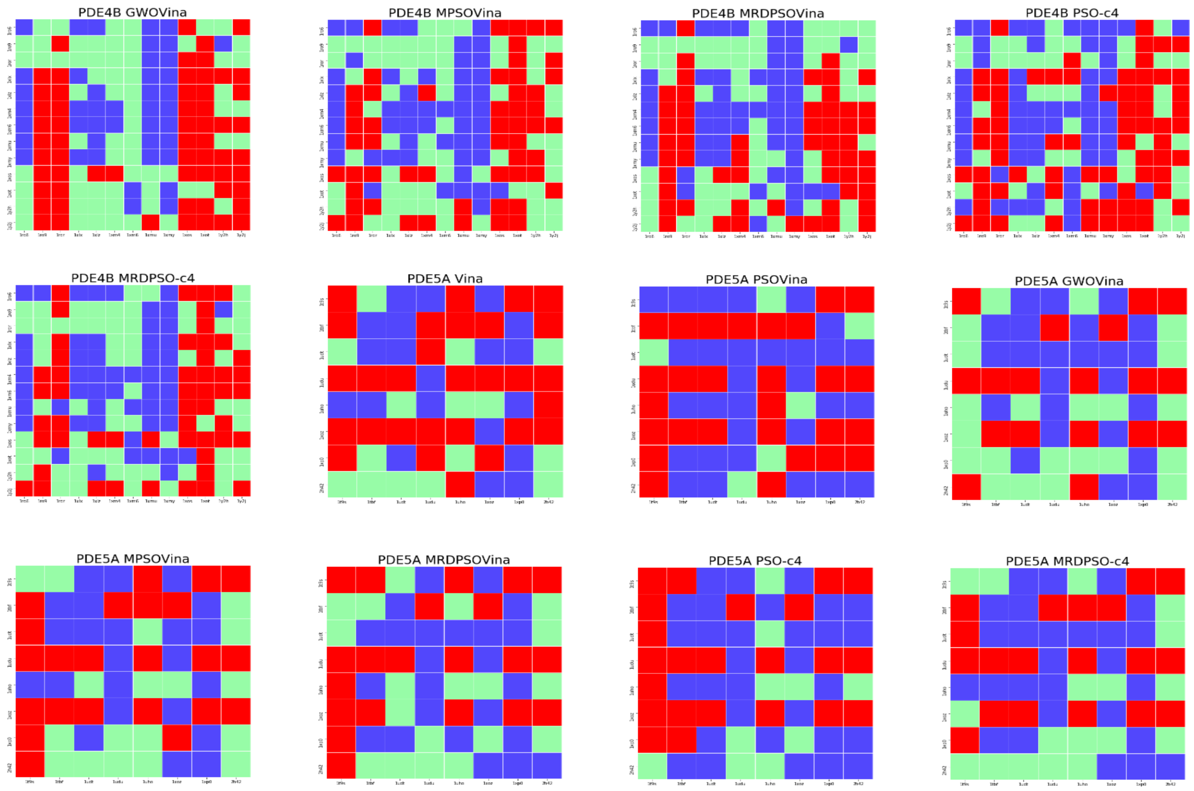

Figure S2. The cross-docking comparison of Vina and Vina-based programs. The cross-docking results were divided into failed sampling (red), failed scoring (green) and successful docking (blue).
